# LGMN promotes crosstalk between macrophages and fibroblasts in pulmonary fibrosis: a potential therapeutic target

**DOI:** 10.3389/fimmu.2026.1789907

**Published:** 2026-04-13

**Authors:** Yuechong Xia, Fang Zhou, Boyue Ma, Haixia Gong, Mengyang Jing, Liping Dai, Songyun Ouyang

**Affiliations:** 1 Department of Respiratory and Critical Care Medicine, the First Affiliated Hospital of Zhengzhou University, Zhengzhou, China; 2 Department of Clinical Pharmacy, The First Affiliated Hospital of Xinxiang Medical University, Weihui, China; 3 Henan Institute of Medical and Pharmaceutical Sciences & Henan Key Medical Laboratory of Tumor Molecular Biomarkers, Zhengzhou University, Zhengzhou, China

**Keywords:** cell interaction, LGMN, macrophage, pulmonary fibrosis, TGF-β signal

## Abstract

**Background:**

Macrophages play a crucial role in the progression of idiopathic pulmonary fibrosis (IPF). This study aims to identify a predictive signature based on macrophage-related genes to forecast patient prognosis and uncover potential therapeutic targets for IPF.

**Methods:**

We analyzed single-cell transcriptomic and microarray data from the GEO database, exploring cellular variations in healthy controls, COPD, and IPF patients. CellChat and Monocle were utilized for analyzing cell interactions and pseudotime trajectories, respectively. Bioinformatics was used to identify differentially expressed genes, leading to the development of a gene signature via multivariate Cox regression, which was validated using ROC curves and an external dataset. The biological function of LGMN was investigated through *in vivo* and *in vitro* experiments.

**Results:**

We observed a significant increase in monocyte-derived macrophages (MDMs) in patients with IPF, which negatively correlated with lung function. In IPF patients, interactions between macrophages and fibroblasts, as well as myofibroblasts, were both more frequent and intense compared to those observed in controls. Notably, the TGF-β1 signaling pathway was significantly activated in IPF, particularly within MDMs and myofibroblasts, leading to increased extracellular matrix (ECM) activity. We developed a gene signature associated with MDMs, which serves as an independent prognostic tool for IPF patients. *In vitro* experiments demonstrated elevated levels of LGMN in M2 macrophages, co-localizing with CD206 in fibrotic lung tissue. Treatment with RR-11a, a LGMN inhibitor, reduced TGF-β1 secretion from M2 macrophages, thereby diminishing communication between macrophages and fibroblasts and alleviating bleomycin-induced pulmonary fibrosis in mice.

**Conclusions:**

Our research establishes a gene signature associated with MDMs, which may aid clinicians in the personalized management of IPF. Additionally, we identify LGMN as a promoter of interaction between M2 macrophages and fibroblasts, suggesting its potential as a therapeutic target for IPF treatment.

## 1 Introduction

Idiopathic pulmonary fibrosis (IPF) is a chronic lung disease characterized by progressive scarring, destruction of alveoli, reduced lung compliance, and impaired gas exchange, ultimately leading to respiratory failure or death (1). The median survival following an IPF diagnosis typically ranges from 2 to 3 years (2). Despite advancements in research and treatment over the past few decades, significant improvements in patient prognosis have remained elusive (3, 4). Current investigations into IPF primarily focus on elucidating new mechanisms and identifying potential therapeutic targets.

Single-cell transcriptomics (scRNA-seq) represents a groundbreaking high-throughput sequencing technique that enables the analysis of gene expression at the individual cell level. This methodology reveals previously unrecognized cellular diversity across various biological contexts and diseases (5, 6). Unlike traditional bulk RNA sequencing, which averages gene expression across many cells, scRNA-seq captures specific expression profiles in distinct cell types, allowing for the identification of unique subpopulations and cellular states (5). This approach is particularly valuable for studying rare cell types, which is critical for understanding tumor microenvironments and autoimmune disorders (7). Furthermore, scRNA-seq facilitates the tracking of gene expression changes over time, illuminating cellular functions throughout the progression of diseases (8). Research employing this technique has identified abnormal cell clusters in conditions such as cancer and Alzheimer’s disease, enhancing our understanding of disease mechanisms (9). Additionally, it highlights early gene expression alterations that may reveal underlying processes. Genes that are specifically expressed in particular cell types can serve as promising therapeutic targets, thereby advancing personalized medicine (10).

In this study, we conducted a comprehensive analysis of single-cell data, revealing a variety of cellular subpopulations in IPF. We found that MDMs exhibited a negative correlation with lung function in IPF patients. Moreover, cell communication analysis demonstrated increased interactions between MDMs and myofibroblasts within the TGF-β signaling pathway. Spatial transcriptomics further revealed that MDMs and myofibroblasts co-localized. Leveraging markers linked to MDMs, we devised a prognostic model that incorporates two genes: *LGMN* (legumain, which encodes a cysteine protease) and *EMP1* (epithelial membrane protein 1, which participates in the apoptotic process and bleb assembly). Furthermore, we validated the critical role of LGMN in facilitating the interaction between M2 macrophages and fibroblasts through comprehensive *in vivo* and *in vitro* functional assays. In summary, our multi-omics study provides novel insights into the mechanisms and potential treatment strategies for IPF, suggesting that targeting LGMN may represent a promising therapeutic approach for this challenging disease.

## 2 Materials and methods

### 2.1 Publical data acquisition and processing

We obtained the single-cell RNA sequencing data from GSE136831 via the GEO database (11). Data analysis was performed using the R package ‘Seurat’ (version 4.1.1) (12), following the methodology outlined in a previous study (13). The scRNA-seq data was normalized using the Seurat ‘NormalizedData’ function with its default parameters. Highly variable genes were detected using the ‘FindVariableFeatures’ function in Seurat, configured with ‘selection.method = vst’ and ‘nfeatures= 2000’. The ‘ScaleData’ function was used to scale the data. For reducing dimensions, the ‘RunPCA’ function was applied. To correct batch effects among samples, the ‘harmony’ package (v0.1.1) with the ‘RunHarmony’ function was utilized (14). The Seurat ‘RunUMAP’ function was used to achieve non-linear dimensionality reduction, and the ‘findallmarkers’ function analyzed the expression of marker genes for major cell types. Differentially expressed genes (DEGs) were identified using criteria that included a log2-fold change exceeding 0.5, a p-value less than 0.01, and a minimum percentage above 0.5.

The gene expression matrix and related clinical data for the GSE70866 dataset were sourced from GEO (11). Samples lacking complete clinical data were excluded, leaving a final dataset consisting of 20 healthy donors and 62 IPF patients from Freiburg, as well as 50 patients from Siena and 64 from Leuven (15). The limma package (version 3.58.1) in R was used to perform quantile normalization (16). To correct for batch effects, the SVA package in R (version 3.50.0) was utilized (17). Annotation of probes was done using platform files, and for genes represented by multiple probes, we calculated the average expression level for analysis. The identification of DEGs between IPF patients and healthy donors was conducted using the limma package, with a significance threshold of adjusted p value<0.05 and log2|*fold* change| > 1.

### 2.2 Cell–cell interaction analysis by Cellchat

Cellchat is an open repository that includes curated data on receptors, ligands, and their interactions, which can be used to search for specific ligands or receptors (18). For this study, we used the Cellchat database to explore cell communication. We first extracted the gene expression matrix and metadata from the integrated scRNA-seq data, and then utilized Cellchat’s statistical analysis method to investigate cell-to-cell communication.

### 2.3 Spatial transcriptomics analysis

Cell2Location is a computational method designed to infer the spatial localization of cell types in tissue samples from single-cell RNA sequencing (scRNA-seq) data (19). The core principle of Cell2Location is the integration of spatial transcriptomics information with single-cell gene expression profiles to predict the distribution of various cell types within the spatial context of a tissue. In this study, 10X Visium Spatial Transcriptomics (ST) data of IPF patients were downloaded from EMBL-EBI dataset (S-BSST1410), and the cell populations identified by single-cell analysis were mapped onto lung tissue sections using the Cell2Location method to conduct intercellular colocalization analysis.

### 2.4 Digital cytometry using CIBERSORTx

Digital cytometry was carried out using the CIBERSORTx (https://cibersortx.stanford.edu/). First, the GSE136831 dataset was used to generate a single-cell reference sample matrix by using findmarkers(). Then the sample matrix was uploaded to CIBERSORTx and a signature matrix file was generated with default parameters. Prior to deconvolution, the microarray mixture dataset (GSE47460) was analyzed with quantile normalization (20).

### 2.5 Construction of risk model and validation

First, we identified the intersection of marker genes for MDMs and DEGs. Subsequently, we conducted a multivariate stepwise Cox regression to select genes with a p-value less than 0.05 for further modeling analysis. Based on the median of risk scores, the prognostic model divided patients in the training and validation cohorts into high-risk or low-risk groups.

The survivalROC package (v1.0.3.1) was utilized to evaluate the model’s prognostic performance through time-dependent ROC curve analysis, as previously described (21). Moreover, an external validation group was employed to verify the predictive accuracy of the prognostic model. The independent predictive value of the risk model was assessed using univariate and multivariate Cox regression analyses, accounting for confounding factors including baseline age and gender. Finally, using the regplot package, we built a nomogram to predict IPF patient survival probability, with calibration curves used to check its accuracy.

### 2.6 Co-culture of macrophages and fibroblast

*In vitro*, we established an M0-M2 differentiation model using a combination of IL-4 and IL-13 (Novoprotein, China). Raw264.7 cells (Haixing Biosciences, Suzhou, China) were treated with IL-4 and IL-13 for another 48 hours. M2 macrophage markers (CD206) were detected by qRT-PCR. The supernatant from M2 cells was collected by centrifuging the cell culture medium at 1,000 × g for 10 minutes to remove cell debris. NIH3T3 cells (Pu-nuo-sai Life Technology Co. Ltd. Wuhan, China) were then treated with the collected supernatant from M2 cells at a ratio of 1:1 (v/v) for 24 hours, as a control, NIH3T3 cells were incubated with fresh culture medium without supernatant of M2 cells. legumain-specific inhibitor RR-11a purchased from purchased from Ambeed Inc (Shanghai, China). The determination of RR-11a concentrations was performed according to previous study (22). Following the treatment, NIH3T3 cells were subjected to further analysis.

### 2.7 Western blot analysis

Western blot analysis was used to assess protein levels in cells and in lung tissues. After the cells and tissues were harvested, they were fully lysed with medium-strength RIPA lysis buffer and protein concentration was determined using the BCA protein assay kit (Beyotime, Shanghai). The denatured cell lysates underwent electrophoresis on an SDS-PAGE gel, and the proteins were then moved to nitrocellulose membranes. To block the membrane, it was exposed to 5% milk in TBST for an hour, followed by incubation with primary antibodies targeting FN1 (ab2413, Abcam, 1:1000), LGMN (67017-1-PBS, Proteintech, 1:1000), TGF-β1 (81746-2-RR, Proteintech, 1:1000), β-actin (20536-1-AP, Proteintech, 1:1000), AKT (#9272, CST, 1:1000),p-AKT (#4060, CST, 1:1000), mTOR (66888-1-Ig, Proteintech, 1:2500) and p-mTOR (67778-1-Ig, Proteintech, 1:2500) and β-Tubulin (10094-1-AP, Proteintech, 1:1000) at 4°C. The secondary antibodies were left to incubate at room temperature for one hour. Chemiluminescent images were captured using the ECL Chemiluminescence Substrate Kit (Servicebio. China).

### 2.8 RNA extraction and RT qPCR

Total RNA was extracted from cells using the GeneJET RNA Extraction Kit (Thermo Fisher Scientific, Waltham, USA) and then reverse transcribed into cDNA with the Prime Script RT Reagent Kit (Servicebio, China). The Applied Biosystems 7500 Real-Time PCR System was used to perform quantitative PCR (qPCR) with the SYBR Green Master Mix Kit from Servicebio, China. Primer sequences are provided in Additional file 1: **Supplementary Table S1**. Quantification of relative mRNA expression levels was performed using the 2−ΔΔCt method.

### 2.9 Immunofluorescence and colocalization analysis

Double immunofluorescence co-localization was conducted on formalin-fixed, paraffin-embedded (FFPE) mouse lung sections to identify the presence of LGMN, TGF-β1, and MRC1 (mannose receptor C-type 1, a type I membrane receptor that facilitates the endocytosis of glycoproteins by macrophages and serves as a marker for M2 macrophages) in macrophages. Following deparaffinization, rehydration, and antigen retrieval, non-specific binding was inhibited for one hour at room temperature.

Slides were incubated with primary antibodies, then HRP-conjugated secondary antibodies, followed by tyramide signal amplification. Nuclei were counterstained with DAPI, and images were captured using an SP5 Leica confocal microscope. Finally, the relative fluorescence intensity was also calculated with ImageJ software.

### 2.10 Animal model and ethics statement

Animal experiments were conducted in accordance with the guidelines set forth by the Ethics Committee at The First Affiliated Hospital of Xinxiang Medical University. One hundred male mouse, aged twelve weeks, were allocated into five distinct groups. The experimental cohort was administered a 5 mg/kg dose of bleomycin (BLM, MedChemExpress) via intratracheal injection in saline, under anesthesia. The sham group received an equivalent volume of saline alone. For the assessment of legumain inhibition, mice were administered either a vehicle control or the legumain inhibitor RR-11a (source leaf organisms, Shanghai, China) at dosages of 20 mg/kg or 10 mg/kg, delivered daily through intraperitoneal injection, commencing seven days post-BLM administration (23). The positive control group was treated with daily intragastric doses of nintedanib (NTB, 60 mg/kg, Selleck). Twenty-four hours after the final dose in 21 days post-BLM administration, mice were anesthetized with 1% pentobarbital sodium (50 mg/kg, i.p.) and euthanized by cervical dislocation. The survival status of each mouse was observed and measured daily.

### 2.11 Enzyme linked immunosorbent assay

Bronchoalveolar lavage fluid (BALF) was performed using 1.2 mL of cold PBS. The supernatant from the macrophages was collected. Cytokine concentrations in BALF and supernatant were assessed using ELISA. The TGF-β1 (Cat#EK0515) and IL-6 (Cat#EK0411) ELISA kits were bought from Wuhan Boster Biological Technology. According to the kit instructions, the procedure was conducted.

### 2.12 Hematoxylin-eosin and Masson’s trichrome staining of mouse lung tissues

The fixed lungs underwent paraffin embedding, followed by sectioning into 5 μm slices and staining with hematoxylin-eosin (HE) or Masson’s trichrome to assess structural changes, inflammation, and collagen deposition. Images were captured using a digital camera mounted on an upright microscope.

### 2.13 Statistical analysis

In this study, statistical analyses were performed utilizing R software (version 4.0.5) and GraphPad Prism (version 8.0). The results are presented as the mean ± standard error of the mean (SEM). Normality and homogeneity of variance tests were conducted on the measurement data for each group. One-way ANOVA was utilized for data meeting the assumptions of normal distribution and homogeneity of variance. For variables that did not follow a normal distribution, the Wilcoxon or Kruskal–Wallis test was applied. Survival times were estimated using the Kaplan-Meier method, with comparisons executed through the log-rank test. To account for potential confounding variables, multivariate Cox regression models were employed, with a p-value of less than 0.05 considered indicative of statistical significance (*p < 0.05, **p < 0.01, ***p < 0.001, ****p < 0.0001).

## 3 Results

### 3.1 The heterogeneous cellular composition between IPF and health donors

**Figure 1:**
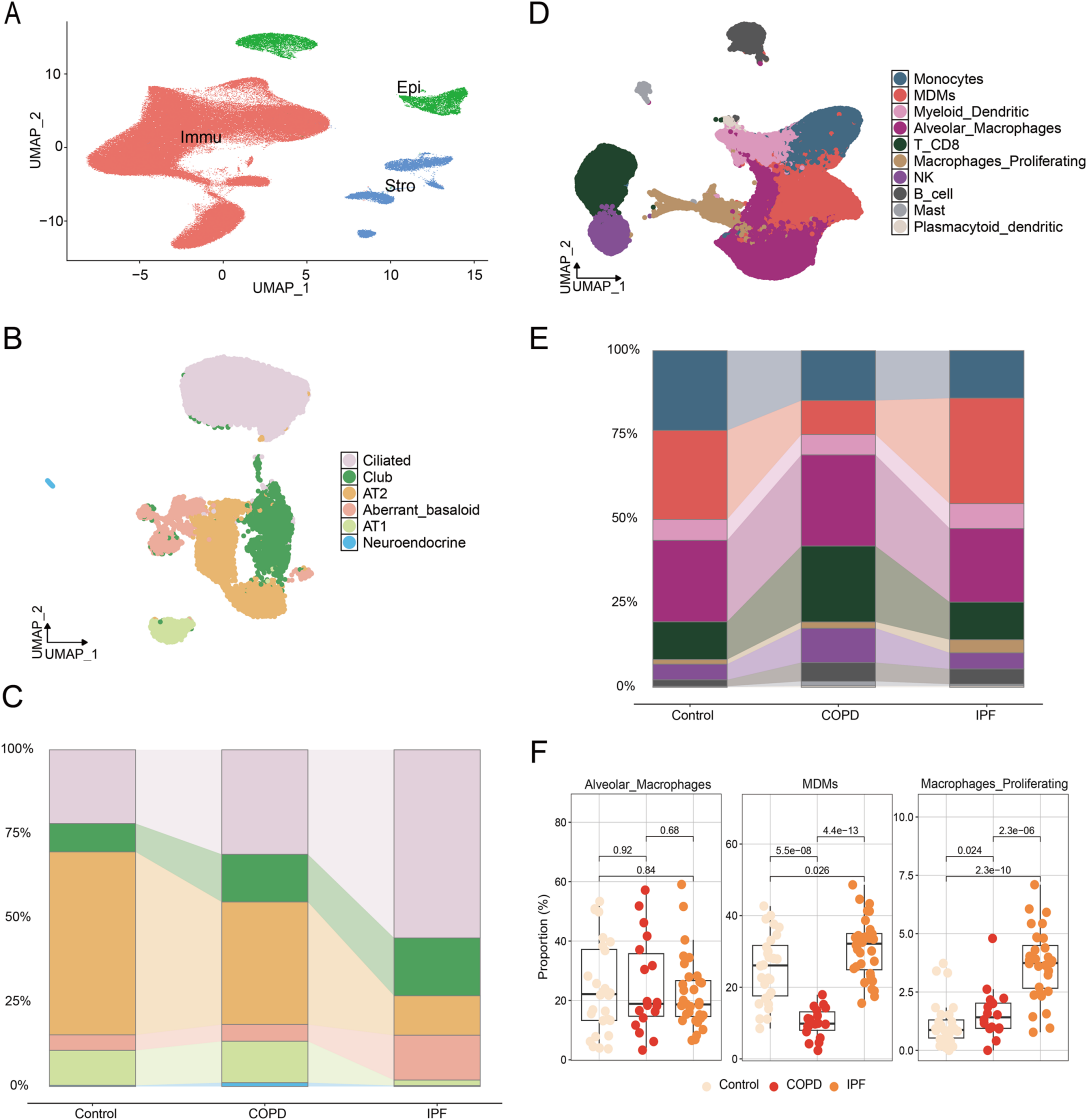
Single cell transcriptome analysis of epithelial cells and immune cells in IPF. **(A)** UMAP of all single-cell transcriptomes clustered by main cell type; **(B)** UMAPs of epithelial single-cell transcriptomes color-coded by cell subtypes; **(C)** Quantification of epithelial cell subtypes per tissue type; **(D)** UMAPs of all immune single-cell transcriptomes color-coded by cell subtypes; **(E)** Quantification of epithelial cell types per tissue type; **(F)** Statistics of macrophage proportions in per tissue type.

After integrating and conducting quality control on the data, we included 201,538 cells for further analysis. Major cell clusters were identified and annotated based on the expression of classical marker genes. The principal cell clusters were classified into epithelial cells (Epi: *EPCAM, CDH1, KRT7*), immune cells (Immu: *PTPRC*), and mesenchymal cells (Stro: *PECAM1, ACTA2, VWF*; **Figure 1A**; **Supplementary Figure 1A**).

To examine interpatient variability in the epithelial and immune cell compartments, epithelial and immune cells were subsetted and reclustered separately for further analysis. We defined six epithelial cell subclusters using specific marker genes (**Figure 1B**; **Supplementary Figure 1B**). Notably, we observed a significant decrease in the proportion of alveolar epithelial cells, while the proportion of non-alveolar epithelial cells increased, which is characteristic of pulmonary fibrosis (**Figure 1C**). Additionally, we distinguished ten immune cell subclusters based on individual marker genes (**Figure 1D**; **Supplementary Figure 1C**). Importantly, we found that the proportion of monocyte-derived macrophages (MDMs) was significantly elevated in IPF, suggesting they may play a crucial role in the pathophysiology of pulmonary fibrosis (**Figures 1E, F**).

**Figure 2:**
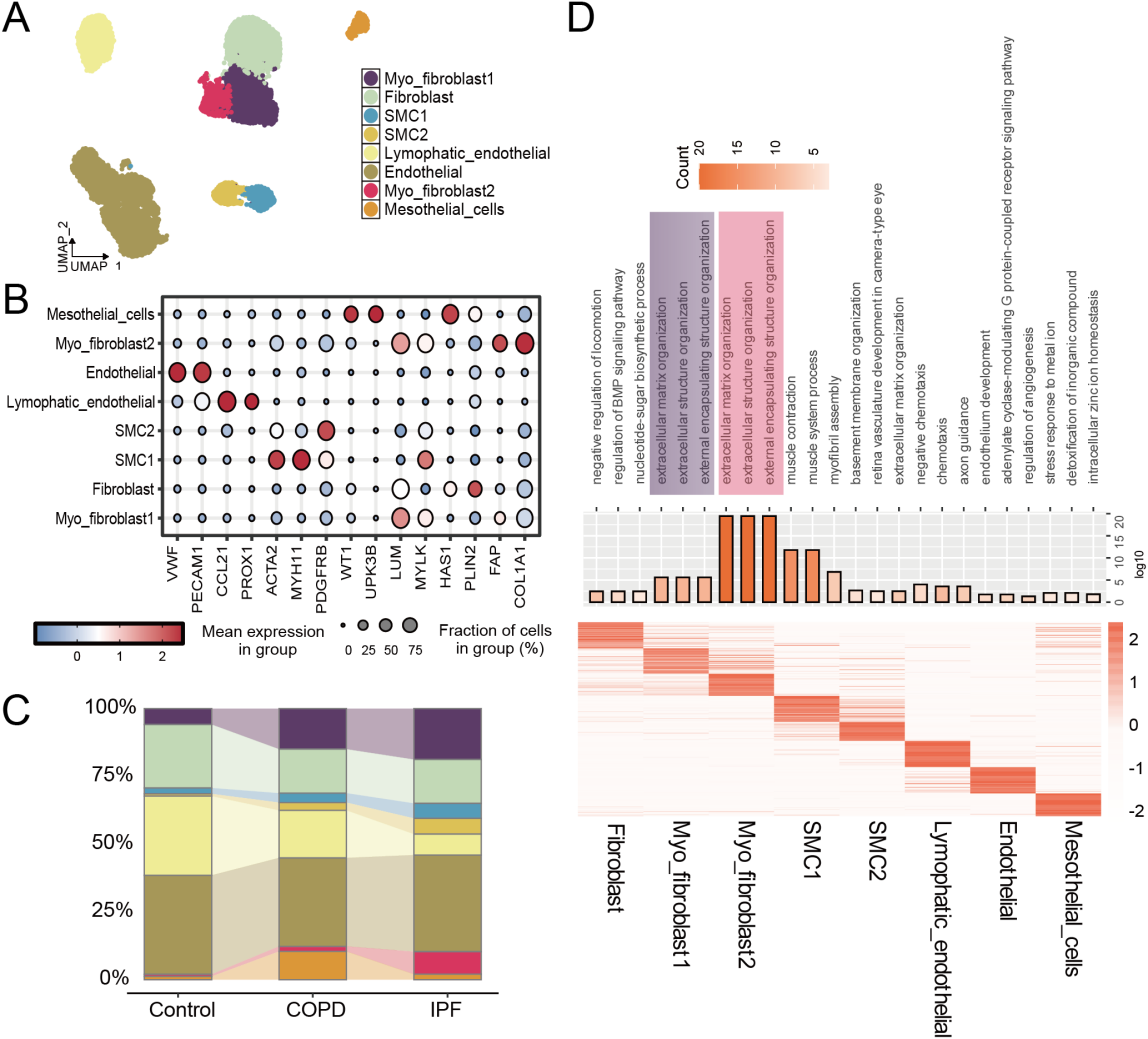
Single cell transcriptome analysis of mesenchymal cells in IPF. **(A)** UMAPs of all mesenchymal single-cell transcriptomes color-coded by cell type; **(B)** Average gene expression of marker genes for mesenchymal cell clusters; **(C)** Quantification of mesenchymal cell types per tissue type; **(D)** GO enrichment analyses of the top 20 upregulated genes for each cell type, highlighting representative GO terms (Top).

In the context of lung fibrosis, primarily characterized by interstitial lung disease, we conducted a detailed analysis of mesenchymal cells and identified eight subpopulations based on cell-specific markers (**Figures 2A, B**). Compared to normal controls, the proportion of fibroblasts decreased, while myofibroblasts—particularly subtype 2—significantly increased (**Figure 2C**). Using the “findAllMarkers” function, we established specific gene profiles for each subpopulation and performed GO analysis, revealing the top three enriched GO terms for each group. The results indicated that myofibroblast subtype 2 may possess enhanced capabilities for extracellular matrix generation (**Figure 2D**).

### 3.2 The intercellular communication between MDMs and myofibroblast

**Figure 3:**
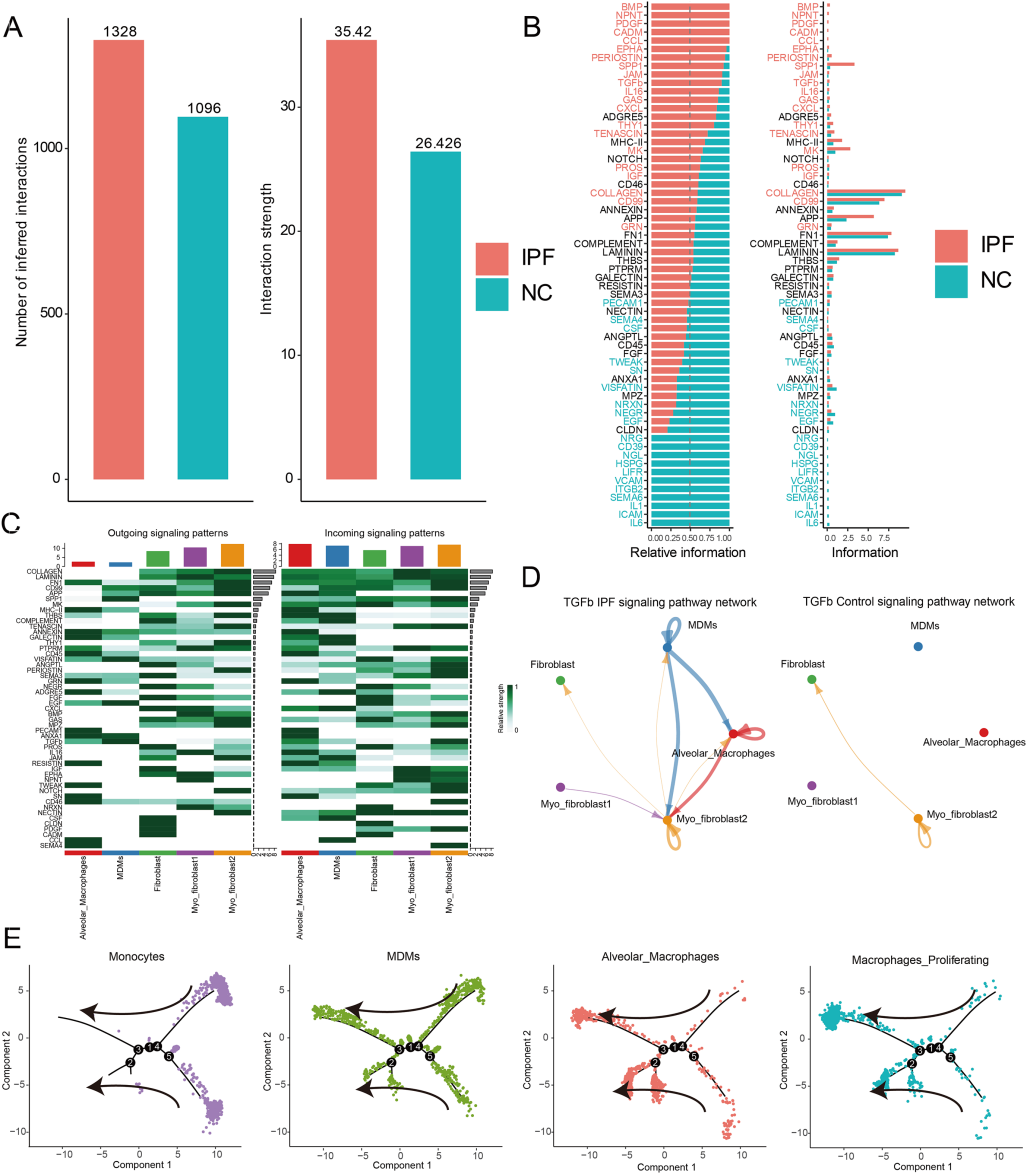
Inference, analysis and visualization of cellular communication networks and pseudotime trajectory analysis via CellChat and Monocle2. **(A)** Histogram shows the difference of cell communication number and strength between macrophages and fibroblast in IPF and health control; **(B)** CellChat analysis indicating alterations in cell-cell communication between IPF and health control. An increase in IPF is represented in red, while blue denotes a decrease in IPF; **(C)** Heatmap shows the strength of each signal pathway network for each cluster with both incoming and outgoing signaling patterns of IPF; **(D)** Circle diagram of cell communication number and weight difference between IPF (Left) and health control (Right); **(E)** Single-cell trajectories reveal developmental relationships of monocytes and macrophages.

We employed CellChat to analyze the changes in cell-cell communication between macrophages and fibroblasts in IPF compared to normal controls. Our analysis revealed a significant increase in both the number and intensity of cell interactions in IPF (**Figure 3A**), highlighting substantial alterations in the communication networks between the two groups. We identified specific communication pathways—namely TGF-β1, SPP1, and BMP—that were highly activated in IPF (**Figure 3B**), consistent with previous studies linking these pathways to pulmonary fibrosis (PF). To further elucidate the input and output signaling patterns among these cell clusters, we utilized a heatmap to illustrate potential signaling pathways. Results indicated that TGF-β1 primarily originated from MDMs and alveolar macrophages (AMs), while the recipient cells were predominantly myofibroblast subtype 2 (**Figures 3C, D**). We found that the TGF-β1 pathway mediated communication between MDMs and AMs. Using pseudotime analysis, we mapped the differentiation trajectory of monocytes into AMs and observed that MDMs were evenly distributed throughout this process. This suggests that they may represent an intermediate stage in the transition from monocytes to AMs (**Figure 3E**). Notably, TGF-β1 signaling was identified as a key pathway facilitating communication between these cell types, supporting previous findings that TGF-β1 plays a crucial role in the promotion of alveolar macrophage formation.

### 3.3 Multiomics analysis revealed the spatial positions of MDMs and myofibroblasts in IPF

**Figure 4:**
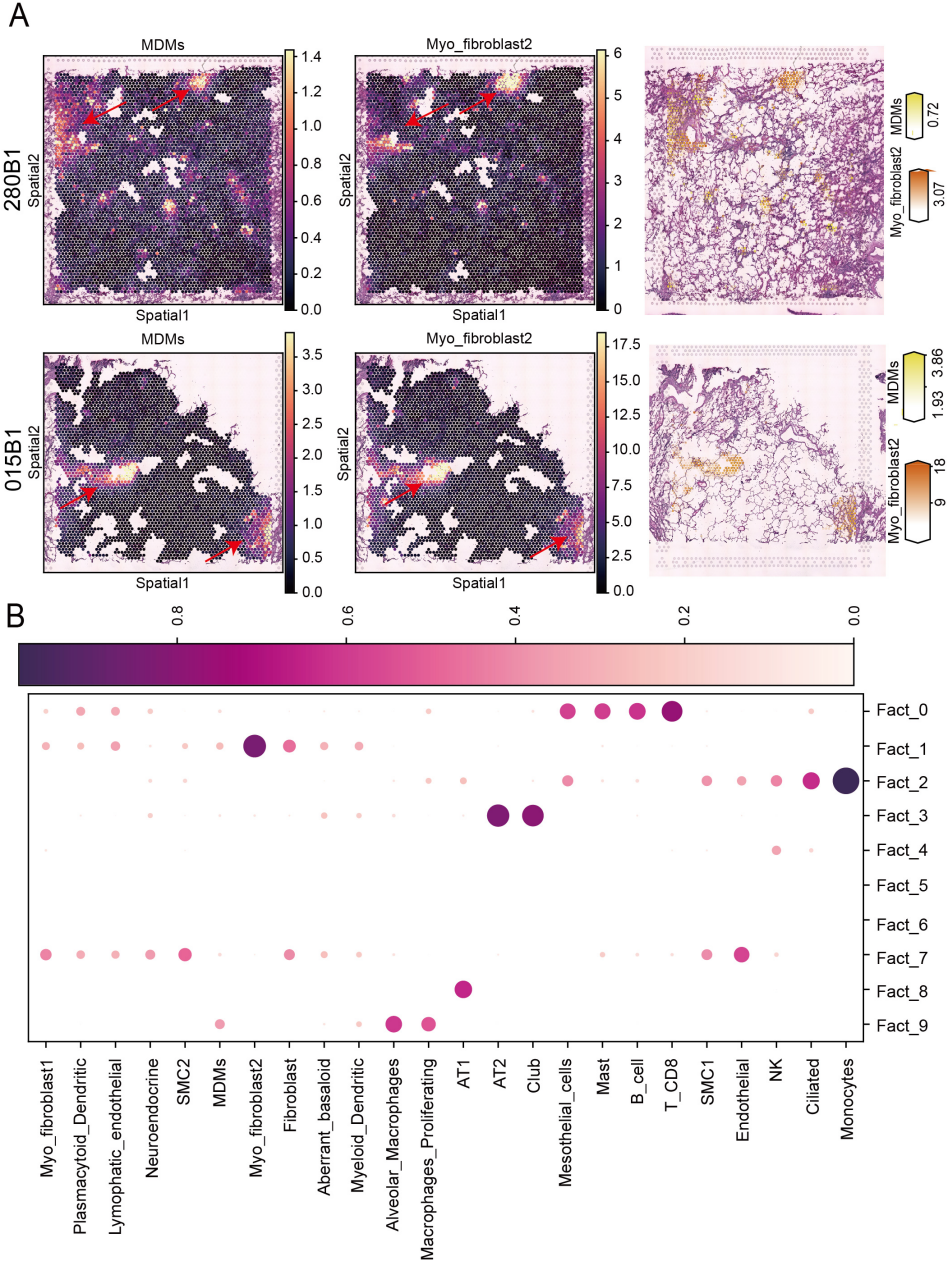
Multiomics analysis revealed spatial positioning of MDMs and myofibroblast type 2 in IPF. **(A)** Spatial images depicting the cell abundance estimated by cell2location for MDMs and myofibroblast type 2 cells on representative IPF tissues; **(B)** Identification of cell compartments using NMF. A dot plot of the estimated NMF weights of cell types (columns) across NMF components (rows), which correspond to cellular compartments.

Although comprehensive single-cell analyses have highlighted intercellular connections between MDMs and myofibroblasts, the specific spatial distribution of these cells requires further exploration. To investigate this, we downloaded spatial transcriptomic data from IPF samples and integrated it with scRNA-seq data using a deconvolution approach. Our analysis revealed colocalization of MDMs and myofibroblast subtype 2 in IPF tissues, indicating that these cells are spatially close to each other (**Figure 4A**). Additionally, non-negative matrix factorization (NMF) decomposition identified factors that characterize spatial cell type abundance profiles, capturing co-localized cell types, including MDMs, fibroblasts, myofibroblast subtype 2, and aberrant basaloid cells (**Figure 4B**). These findings confirm the close spatial proximity of MDMs and myofibroblast subtype 2 in IPF tissues.

### 3.4 MDMs was negatively associated with lung function of IPF

**Figure 5:**
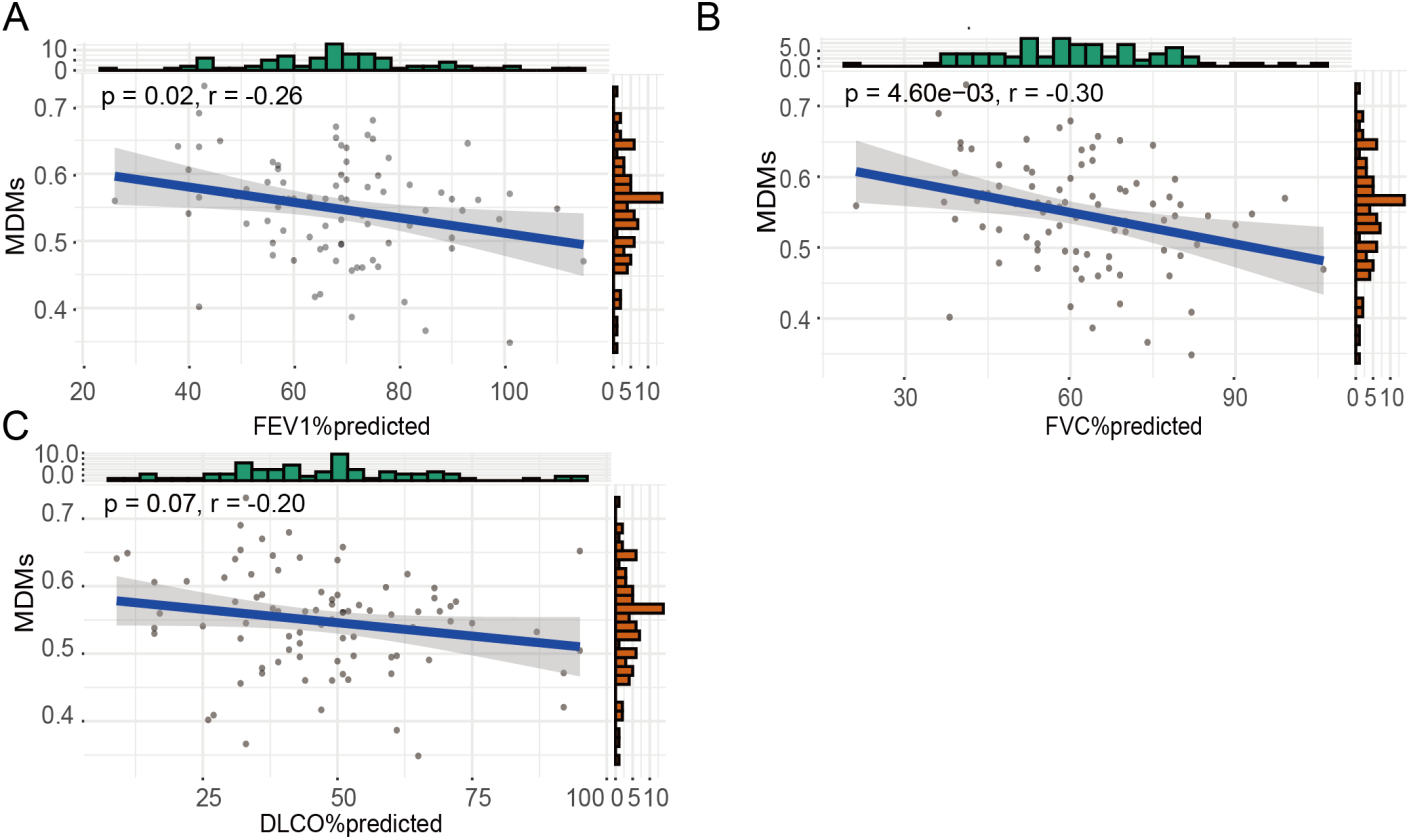
Infiltration of MDMs was negatively associated with lung function of IPF. **(A)** Correlations between abundance of MDMs and FEV1%predicted; **(B)** Correlations between abundance of MDMs and FVC%predicted; **(C)** Correlations between abundance of MDMs and DLCO%predicted.

To further elucidate the critical role of MDMs in lung fibrosis, we evaluated their infiltration levels in fibrotic lung tissue and conducted a joint analysis with the patients’ pulmonary function metrics. We utilized CIBERSORTx for digital cytometry to assess MDMs infiltration levels from bulk RNA sequencing data. The results demonstrated a significant negative correlation between MDMs infiltration and lung function, particularly with percent predicted forced expiratory volume in one second (FEV1% predicted, *p* = 0.02) and percent predicted forced vital capacity (FVC% predicted, *p* = 4.60e−03, **Figures 5A, B**). Additionally, there was a trend suggesting an inverse correlation between MDM infiltration and percent predicted diffusion lung capacity for carbon monoxide (DLCO% predicted), although it did not reach statistical significance (*p* = 0.07, **Figure 5C**).

### 3.5 Construction of prognostic risk model

**Figure 6:**
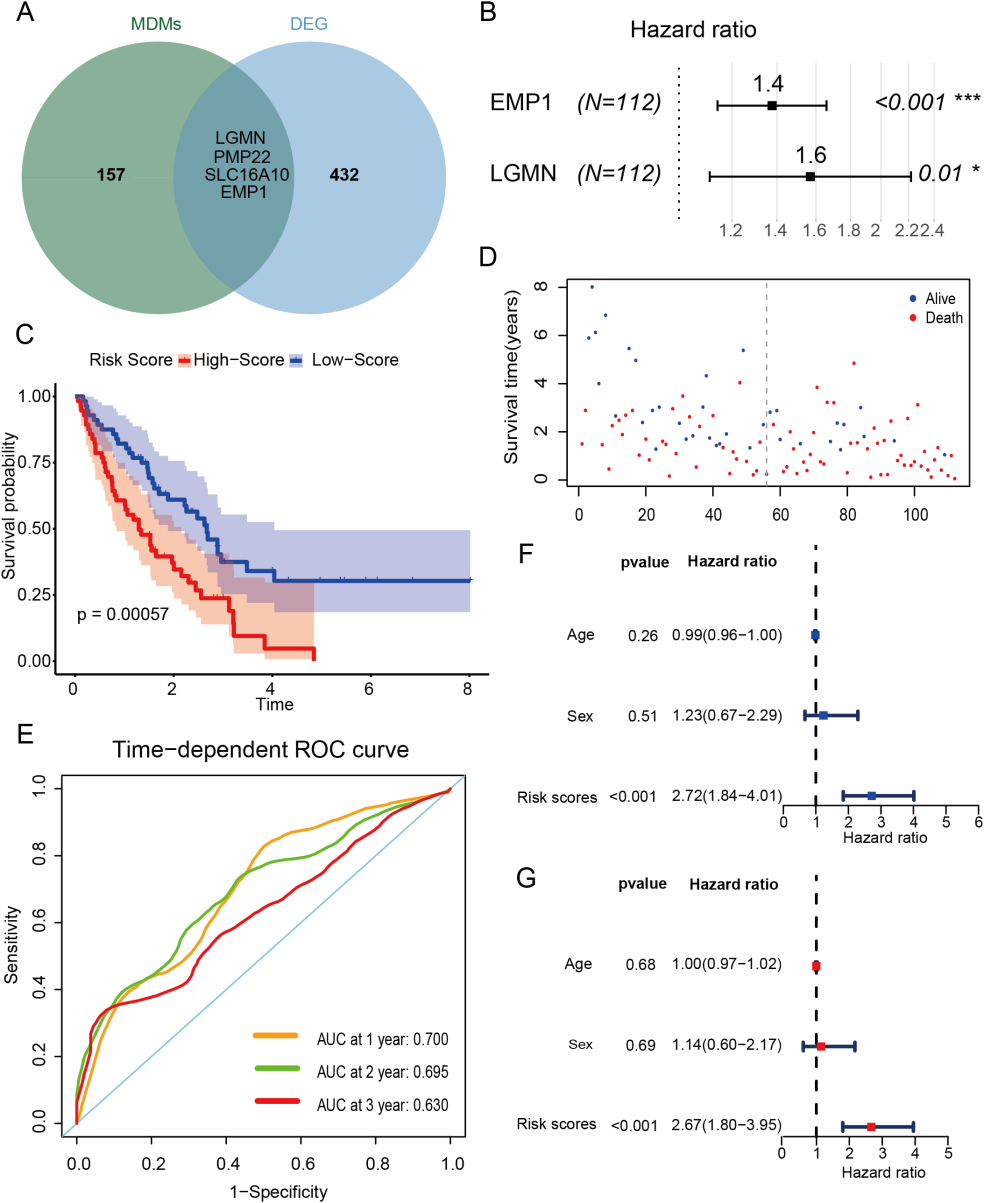
Construction of an MDMs based outcome prediction model. **(A)** Wayne diagram displaying the intersect of MDMs specific marker genes and DEGs; **(B)** Two genes were selected by multivariate Cox regression analysis with stepwise regression; **(C)** The curve of survival status distribution in training set; **(D)** distribution of risk scores based on the survival status of patients with risk scores in training set; **(E)** ROC curves of the risk score for 1-year survival, 2-year survival and 3-year survival in training set; **(F)** The univariate Cox regression analyses in training set; **(G)** The multivariate Cox regression analyses in training set. *p < 0.05, ***p < 0.001.

Using the findAllMarkers function, we identified specific marker genes for MDMs and intersected them with DEGs from bulk RNA sequencing (**Supplementary Files S1, S2**). This analysis pinpointed four essential genes: *LGMN*, *EMP1*, *PMP22* (peripheral myelin protein 22, which encodes an integral membrane protein critical for myelin formation in the peripheral nervous system), and *SLC16A10* (solute carrier family 16 member 10, which encodes a member of a family of plasma membrane amino acid transporters that facilitate the Na(+)-independent transport of aromatic amino acids across the plasma membrane) (**Figure 6A**).We selected patients from Freiburg and Siena for the training set (n=112), while patients from Leuven were used as the external testing set (n=64). *EMP1* and *LGMN* were then chosen to construct a prognostic model using multivariate stepwise Cox regression (**Figure 6B**). The resulting risk score (RS) is calculated as follows: RS = 0.32 × expression of *EMP1* + 0.45 × expression of *LGMN*.

### 3.6 Two-gene signature serves as a risk factor for patients with IPF

In the training set, 112 patients were divided into high-risk and low-risk groups based on their median risk scores. Kaplan-Meier analysis revealed that the high-risk group had significantly poorer clinical outcomes (**Figures 6C, D**). AUC analysis further confirmed the model’s robust predictive capability for overall survival in IPF, with AUC values of 0.7, 0.695, and 0.63 for survival at 1, 2, and 3 years, respectively (**Figure 6E**). Additionally, both univariate and multivariate Cox proportional hazards analyses identified the 2-gene signature as an independent prognostic factor (**Figures 6F, G**).

**Figure 7:**
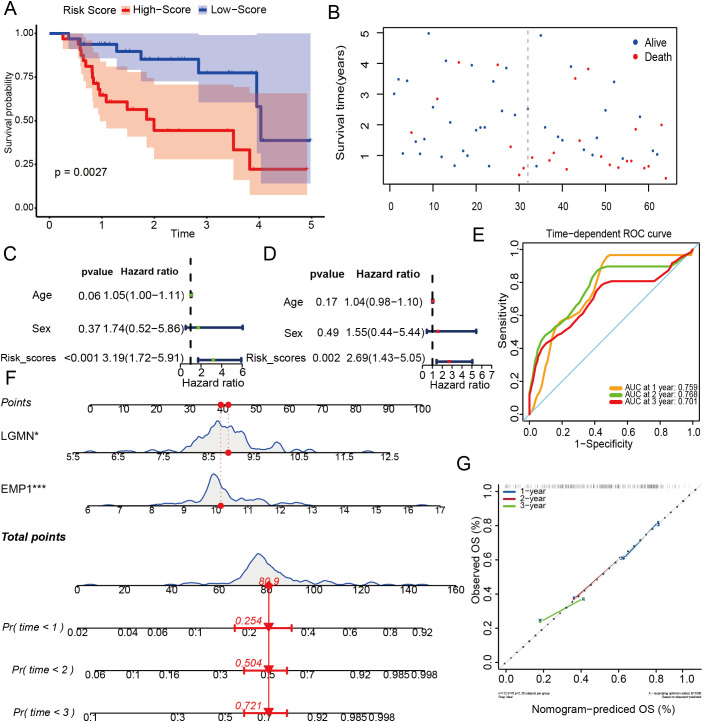
External validation of the prediction model. **(A)** The curve of survival status distribution in external test set; **(B)** Distribution of risk scores based on the survival status of patients with risk scores in training set; **(C)** The univariate Cox regression analyses in training set; **(D)** The multivariate Cox regression analyses in training set; **(E)** ROC curves of the risk score for 1-year survival, 2-year survival and 3-year survival in training set; **(F)** Nomogram model for predicting IPF patients; **(G)** The calibration curve for the Nomogram model. *p < 0.05, ***p < 0.001.

In the external test set, 64 patients were classified into high- and low-risk categories based on median scores. Kaplan-Meier analysis revealed that the high-risk group experienced significantly worse clinical outcomes compared to the low-risk group (**Figures 7A, B**). Both univariate and multivariate Cox proportional hazards analyses identified the 2-gene signature as an independent risk factor (**Figures 7C, D**). The AUC analysis confirmed the model’s robust predictive capability for overall survival in IPF, yielding AUC values of 0.759, 0.768, and 0.701 for survival at 1, 2, and 3 years, respectively (**Figure 7E**). Finally, a nomogram was developed for clinical use based on the 2-gene signature, and a calibration curve was created to assess its predictive accuracy. The calibration chart demonstrated that the predicted survival probabilities for 1, 3, and 5 years closely matched the observed data (**Figures 7F, G**).

### 3.7 Inhibition of LGMN suppressed fibroblast activation mediated by macrophages

**Figure 8:**
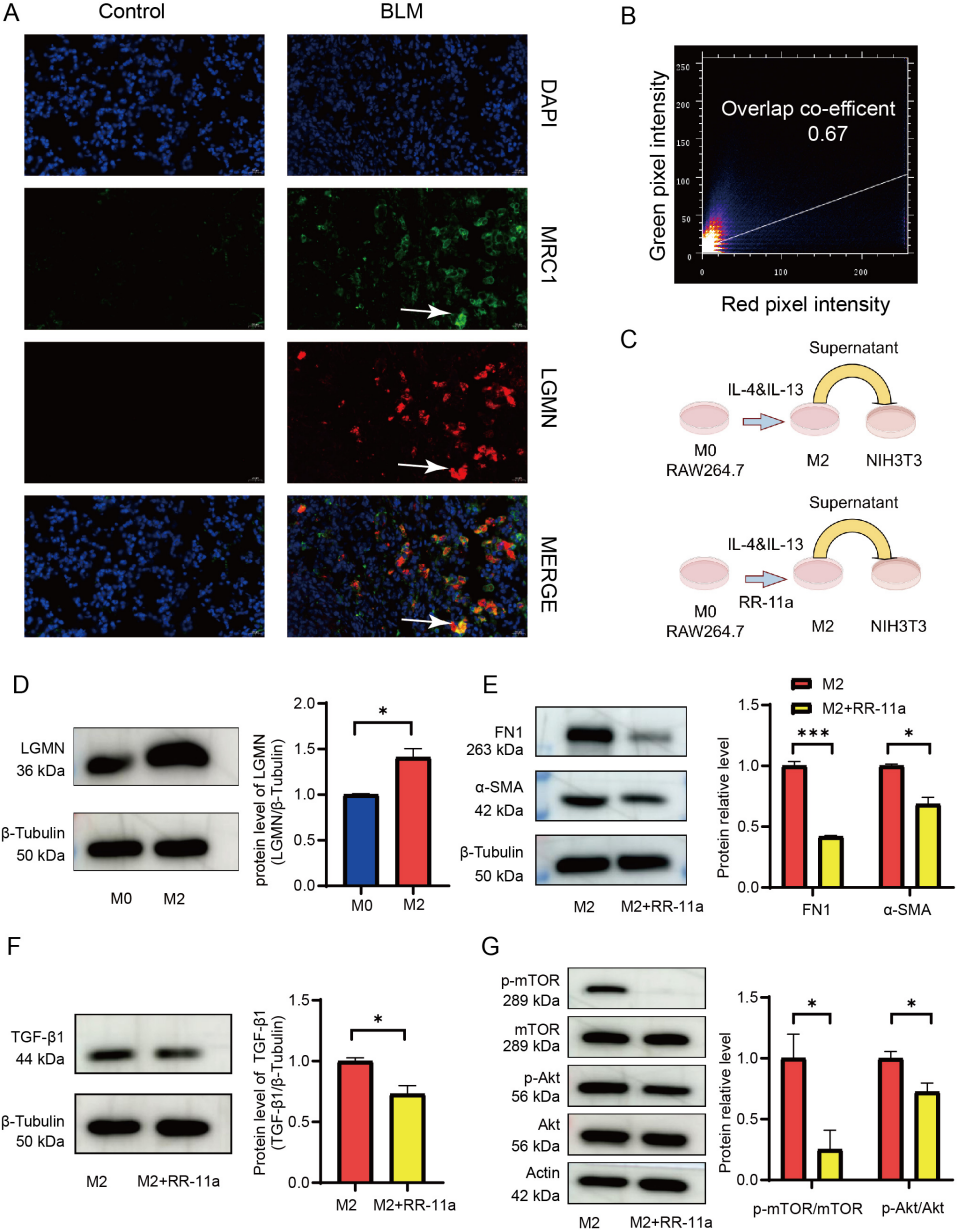
LGMN is primarily expressed in M2 macrophages and promotes the secretion of TGF-β1. **(A)** Double immunofluorescence showed that LGMN was colocalized with M2 macrophage marker MRC1, white arrows indicate colocalization. **(B)** Graphical representation of colocalization analysis based on the overlap rate on each cell. **(C)** Flow chart of the *in vitro* experiments. **(D)** Representative western blots and quantification of LGMN in M2 macrophages (n=3). **(E)** Representative western blots and quantification of FN1 and α-SMA in NIH3T3 cells cultured with medium from M2 macrophages that were pretreated with or without RR-11a (n=3). **(F)** Representative western blots and quantification of TGF-β1 in M2 macrophages treated with or without RR-11a (n=3). **(G)** Representative western blot analyses of p-Akt/Akt and p-mTOR/mTOR expression levels in M2 macrophages treated with or without RR-11a (n=3). *p < 0.05, ***p < 0.001.

According to the risk scoring model, *LGMN* exhibits a higher risk coefficient. Consequently, we have decided to pursue further validation and analysis of *LGMN*. To confirm co-localization between LGMN and M2 macrophages, we conducted MIF on lung tissue from mouse. The results demonstrated co-localization of LGMN and MRC1, with an overlap coefficient of 0.68 (**Figures 8A, B**). A brief overview of the experimental workflow is illustrated in **Figure 8C**. RAW264.7 cells expressed high levels of *Mrc1* and *Tgfb1* after treatment with IL-4 and IL-13, confirming their differentiation into M2 macrophages (**Supplementary Figures 2A, B**). Western blot analysis showed a significant increase in LGMN expression in these cells (**Figure 8D**). Co-culturing NIH3T3 fibroblasts with the supernatant from M2 macrophages resulted in marked fibroblast activation (**Supplementary Figure 2C**). However, co-culturing NIH3T3 cells with supernatant from M2 macrophages pretreated with RR-11a significantly suppressed this activation, underscoring the critical role of LGMN in this process (**Figure 8E**). Additionally, we demonstrated that LGMN inhibition reduces TGF-β1 secretion from M2 macrophages (**Figure 8F**; **Supplementary Figure 2D**). The Akt/mTOR signaling pathway is known to regulate differentiation (24, 25). To elucidate the molecular mechanism by which LGMN operates, we examined the activation status of key components in the Akt/mTOR pathway using Western blot analysis. As shown in **Figure 8G**, the phosphorylation of critical proteins, including Akt and mTOR, was decreased in M2 macrophages pretreated with RR-11a. These findings indicate that LGMN promotes M2 macrophage differentiation via the Akt/mTOR signaling pathway.

### 3.8 Pharmacological blockade of LGMN ameliorated PF by BLM

**Figure 9:**
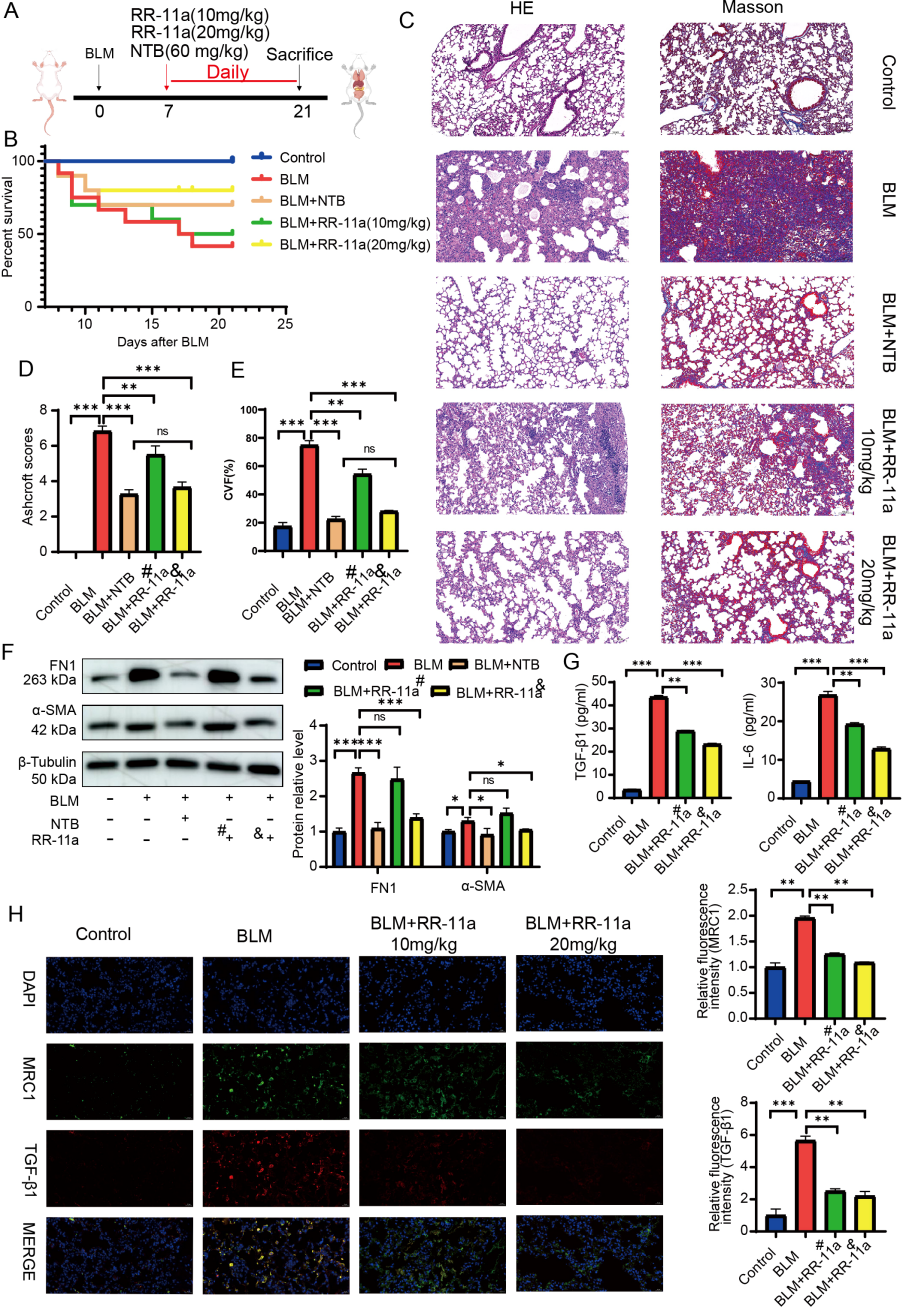
Pharmacological blockade of LGMN ameliorated PF by BLM. **(A)** Experimental flow chart of the animal study. **(B)** Survival curves of mice in each group. (n = 12). **(C)** Representative images for **(H, E)** (left) and Masson staining (right) of the severity of lung fibrosis in mice between five groups. **(D)** Quantification of fibrosis by Ashcroft score (n=3). **(E)** Quantitative analysis of the collagen volume fraction of different groups (n=3). **(F)** Western blot analysis of FN1 and α-SMA expression in the lung homogenate of mice from five groups (n=3). **(G)** Levels of TGF-β1and IL-6 in the BALF were analyzed using ELISA (n=5). **(H)** Immunofluorescence staining and quantitative analysis of MRC1 and TGF-β1 in the lung sections from mice (n=3). The nuclei were stained blue by DAPI. ^#^:10mg/kg; ^&^:20mg/kg. *p < 0.05, **p < 0.01, ***p < 0.001, ****p < 0.0001, ns, no significance.

Based on our findings regarding the critical role of LGMN in macrophage-fibroblast interactions, we investigated the potential of LGMN inhibition as a therapeutic strategy for PF. We employed a BLM-induced lung fibrosis mouse model, which is widely used for studying lung fibrosis. A brief overview of the experimental design is presented in **Figure 9A**. Day 7 post-BLM treatment marks the onset of the fibrotic phase, coinciding with the resolution of acute inflammation. We administered intraperitoneal injections of RR-11a on day 7 to assess its anti-fibrotic effects. Daily monitoring revealed that BLM treatment resulted in increased mortality, while co-treatment with RR-11a significantly improved survival rates (**Figure 9B**). Both NTB and RR-11a administration markedly reduced lung fibrosis development as assessed through HE staining and Ashcroft scoring (**Figures 9C, D**), which was further corroborated by Masson trichrome staining (**Figure 9E**). Western blot analysis indicated that RR-11a substantially lowered the protein levels of FN1 and α-SMA (**Figure 9F**). Additionally, RR-11a inhibited the release of IL-6 and the profibrotic factor TGF-β1 in BLM-induced fibrotic lungs, highlighting the pro-inflammatory and profibrotic roles of LGMN (**Figure 9G**). Immunofluorescence staining of M2 macrophages and TGF-β1 expression showed that RR-11a treatment reduced M2 macrophage infiltration and TGF-β1 expression in PF (**Figure 9H**).

## 4 Discussion

Alveolar macrophages and MDMs are critical immune cell populations in lung tissue, linked to fibrosis, inflammation, and immune regulation (26, 27). In this study, we observed that macrophages in IPF exhibit increased interactions with myofibroblasts, which are characterized by heightened ECM activity. Moreover, the infiltration of MDMs negatively correlates with lung function. MDMs may represent an intermediate state in the transition from monocytes to alveolar macrophages, with the TGF-β1 signaling pathway likely playing a crucial role in their formation and function. We developed a predictive model for PF based on MDM-associated genes. Additionally, our *in vitro* experiments demonstrated that inhibiting LGMN effectively reduces TGF-β1 secretion from M2 macrophages and disrupts their communication with myofibroblasts. These findings suggest potential new therapeutic targets for PF.

An increasing number of studies suggest that multicellular interactions play a crucial role in PF (28–30). The observed enhanced interactions between macrophages and myofibroblasts in our study pave the way for deeper investigations into the functional roles of various macrophage subtypes in the progression of IPF. Future research could focus on elucidating the precise mechanisms by which macrophages, particularly MDMs, influence the fibrotic microenvironment. Understanding these interactions at a molecular level may reveal novel targets for intervention. Our study showed that macrophage infiltration, especially that of MDMs, negatively correlates with lung function in patients. Therefore, exploring the molecular mechanisms that regulate MDM infiltration under fibrotic disease conditions could provide new insights for the prevention and treatment of PF. The interplay between macrophages and myofibroblasts may also present an opportunity for combination therapies targeting both cell types, maximizing antifibrotic effects. This strategy could enhance the efficacy of current treatments while minimizing the potential adverse effects associated with therapies that target a single pathway.

LGMN, or Legumain, is a cysteine protease encoded by the human *LGMN* gene, which plays a critical role in various biological processes such as apoptosis, protein processing, and cell signaling (31, 32). In our study, we demonstrated that inhibiting LGMN can reduce the secretion of TGF-β1 from M2 macrophages and disrupt TGF-β-mediated interactions between macrophages and fibroblasts. These findings complement earlier research indicating that LGMN deficiency is linked to downregulation of anti-inflammatory mediators, such as IL-10 and TGF-β1, and upregulation of pro-inflammatory cytokines like IL-1β, TNF-α, IL-6, and IFN-γ (33). Furthermore, LGMN has been shown to contribute to cancer resistance against anti-PD1 immunotherapy by promoting the polarization of macrophages toward the M2 phenotype (34). Additionally, it facilitates the immunosuppressive polarization of macrophages through the activation of the GSK-3β-STAT3 signaling pathway (35). Previous studies have shown that LGMN is associated with cancer metastasis, vascular dissection, and the senescence of tumor cells through its interaction with integrin αvβ3 (36–38). Specifically, LGMN binds to the cell surface integrin αvβ3, which subsequently activates the PI3K/AKT/mTOR signaling pathway (34). In our research, we confirmed that LGMN enhances the secretion of TGF-β1 from M2 macrophages by activating the AKT/mTOR signaling pathway, which aligns with previous findings. Overall, targeting LGMN may represent a promising strategy for the treatment of PF.

We found that MDM levels are significantly higher in PF compared to COPD and health control, underscoring their critical role in progression of PF. Additionally, we observed that the infiltration of MDMs in lung tissues negatively correlates with lung function. In a mouse model of PF, we also discovered that inhibiting LGMN function can reduce the infiltration of M2 macrophages. MDMs exhibit a dynamic role in the development and progression of pulmonary fibrosis (39). Originating from circulating monocytes, they are rapidly recruited to the lungs during inflammation or injury and differentiate into various functional phenotypes influenced by the local microenvironment. In the context of pulmonary fibrosis, MDMs serve a dual role: in the early stages, they promote tissue repair and restoration, but in later stages, they may contribute to chronic inflammation and exacerbate fibrosis (40, 41). The recruitment of monocytes to the lungs is a finely regulated process orchestrated by chemokines, with the CCL2/CCR2 axis being one of the most critical regulatory pathways. This recruitment is crucial in the early stages of fibrosis, as monocytes provide a vital source of precursors for differentiation into either reparative or fibrosis-promoting macrophages (42, 43). For example, the dual CCR2/CCR5 antagonist Cenicriviroc has shown promise in early clinical trials for fibrotic diseases (44–46).

Once monocytes differentiate into macrophages, their functional phenotypes are influenced by the local pulmonary microenvironment, resulting in significant functional diversity that ranges from pro-inflammatory M1 macrophages to pro-repair M2 macrophages (26, 27). In our study, we found that alveolar macrophages are terminal cells in the differentiation trajectory of monocytes and that MDMs may act as intermediates between monocytes and alveolar macrophages, which aligns with previous studies (47, 48). We further discovered that in IPF, the TGF-β signaling pathway between MDMs and alveolar macrophages is significantly enhanced compared to normal controls, suggesting that macrophages may mediate the differentiation of monocyte-derived macrophages through the autocrine secretion of TGF-β1. Single-cell sequencing technologies have demonstrated the differentiation of MDMs into alveolar macrophages in models of bleomycin-induced lung fibrosis and COVID-19-associated pulmonary fibrosis (49, 50). Additionally, it has been shown that TGF-β1 can promote the differentiation of MDMs into alveolar macrophages (51). Therefore, inhibiting LGMN to reduce the autocrine secretion of TGF-β1 by macrophages may weaken the differentiation of monocyte-derived macrophages and exert an anti-fibrotic effect.

This study has several limitations. First, we did not assess the role of LGMN in human macrophages; instead, we utilized a mouse model for our experiments. Although their physiological characteristics are similar to those of humans, there are still significant differences. Consequently, the clinical translation of our findings may face certain challenges. Second, the mechanisms by which LGMN regulates TGF-β1 secretion from M2 macrophages require further experimental investigation, which will be our focus in future research. Additionally, it is worth noting that we did not establish LGMN knockdown models *in vitro* and *in vivo* to further elucidate the function of LGMN, although we employed pharmacological inhibition methods. Future studies will be necessary to validate our conclusions through more detailed and rigorous experimental approaches.

## 5 Conclusion

In summary, we established a two-gene signature based on MDMs that serves as an independent prognostic predictor with excellent prediction efficiency. Our findings indicate that LGMN promotes secretion of TGF-β1 from M2 macrophages and disrupt TGF-β-mediated cellular interactions between macrophages and fibroblasts. Given these results, LGMN may represent a promising therapeutic target for the treatment of pulmonary fibrosis.

## Data Availability

The original contributions presented in the study are included in the article/**Supplementary Material**. Further inquiries can be directed to the corresponding author/s.

## Supplementary material

Supplementary Figure 1 **(A)** Average gene expression of selected marker genes for main cell clusters; **(B)** Average gene expression of selected marker genes for epithelial cell clusters; **(C)** Average gene expression of selected marker genes for immune cell clusters.

Supplementary Figure 2 **(A)** Relative mRNA expression of *Mrc1* in Raw264.7 cells and M2 macrophages; **(B)** Relative mRNA expression of *Tgfb1* in Raw264.7 cells and M2 macrophages; **(C)** supernatant from the M2 cultures induced the activation of NIH3T3 cells. **(D)** The supernatant of M2 cells was collected after treatment with RR-11a and ELISA performed for analysis of TGF-β1 secreted into the medium.

## References

[1] Richeldi L Collard HR Jones MG . Idiopathic pulmonary fibrosis. Lancet. (2017) 389:1941–52. doi: 10.1007/bf03256611. PMID: 28365056

[2] Kim HJ Perlman D Tomic R . Natural history of idiopathic pulmonary fibrosis. Respir Med. (2015) 109:661–70. doi: 10.1016/j.rmed.2015.02.002. PMID: 25727856

[3] Richeldi L du Bois RM Raghu G Azuma A Brown KK Costabel U . Efficacy and safety of nintedanib in idiopathic pulmonary fibrosis. N Engl J Med. (2014) 370:2071–82. doi: 10.1056/nejmoa1402584. PMID: 24836310

[4] Richeldi L Kolb M Jouneau S Wuyts WA Schinzel B Stowasser S . Efficacy and safety of nintedanib in patients with advanced idiopathic pulmonary fibrosis. BMC Pulm Med. (2020) 20:3. doi: 10.1186/s12890-019-1030-4. PMID: 31914963 PMC6951000

[5] Villani AC Satija R Reynolds G Sarkizova S Shekhar K Fletcher J . Single-cell RNA-seq reveals new types of human blood dendritic cells, monocytes, and progenitors. Science. (2017) 356:eaah4573. doi: 10.1126/science.aah4573. PMID: 28428369 PMC5775029

[6] Sun G Li Z Rong D Zhang H Shi X Yang W . Single-cell RNA sequencing in cancer: Applications, advances, and emerging challenges. Mol Ther Oncolytics. (2021) 21:183–206. doi: 10.1016/j.omto.2021.04.001. PMID: 34027052 PMC8131398

[7] Grün D Lyubimova A Kester L Wiebrands K Basak O Sasaki N . Single-cell messenger RNA sequencing reveals rare intestinal cell types. Nature. (2015) 525:251–5. doi: 10.1038/nature14966, PMID: 26287467

[8] Trapnell C . Defining cell types and states with single-cell genomics. Genome Res. (2015) 25:1491–8. doi: 10.1101/gr.190595.115. PMID: 26430159 PMC4579334

[9] Mathys H Davila-Velderrain J Peng Z Gao F Mohammadi S Young JZ . Single-cell transcriptomic analysis of Alzheimer's disease. Nature. (2019) 570:332–7. doi: 10.1038/s41586-019-1195-2. PMID: 31042697 PMC6865822

[10] Zhang L Yu X Zheng L Zhang Y Li Y Fang Q . Lineage tracking reveals dynamic relationships of T cells in colorectal cancer. Nature. (2018) 564:268–72. doi: 10.1038/s41586-018-0694-x. PMID: 30479382

[11] Barrett T Wilhite SE Ledoux P Evangelista C Kim IF Tomashevsky M . NCBI GEO: archive for functional genomics data sets--update. Nucleic Acids Res. (2013) 41:D991–995. doi: 10.1093/nar/gks1193. PMID: 23193258 PMC3531084

[12] Butler A Hoffman P Smibert P Papalexi E Satija R . Integrating single-cell transcriptomic data across different conditions, technologies, and species. Nat Biotechnol. (2018) 36:411–20. doi: 10.1038/nbt.4096. PMID: 29608179 PMC6700744

[13] Wang P Xu S Guo Q Zhao Y . Discovery of PAK2 as a key regulator of cancer stem cell in head and neck squamous cell carcinoma using multi-omic techniques. Stem Cells Int. (2025) 2025:1325262. doi: 10.1155/sci/1325262. PMID: 41311809 PMC12657082

[14] Korsunsky I Millard N Fan J Slowikowski K Zhang F Wei K . Fast, sensitive and accurate integration of single-cell data with Harmony. Nat Methods. (2019) 16:1289–96. doi: 10.1038/s41592-019-0619-0. PMID: 31740819 PMC6884693

[15] Prasse A Binder H Schupp JC Kayser G Bargagli E Jaeger B . BAL cell gene expression is indicative of outcome and airway basal cell involvement in idiopathic pulmonary fibrosis. Am J Respir Crit Care Med. (2019) 199:622–30. doi: 10.1164/rccm.201712-2551oc. PMID: 30141961 PMC6396865

[16] Ritchie ME Phipson B Wu D Hu Y Law CW Shi W . limma powers differential expression analyses for RNA-sequencing and microarray studies. Nucleic Acids Res. (2015) 43:e47. doi: 10.1093/nar/gkv007. PMID: 25605792 PMC4402510

[17] Leek JT Johnson WE Parker HS Jaffe AE Storey JD . The sva package for removing batch effects and other unwanted variation in high-throughput experiments. Bioinformatics. (2012) 28:882–3. doi: 10.1093/bioinformatics/bts034. PMID: 22257669 PMC3307112

[18] Jin S Guerrero-Juarez CF Zhang L Chang I Ramos R Kuan CH . Inference and analysis of cell-cell communication using CellChat. Nat Commun. (2021) 12:1088. doi: 10.1038/s41467-021-21246-9. PMID: 33597522 PMC7889871

[19] Kleshchevnikov V Shmatko A Dann E Aivazidis A King HW Li T . Cell2location maps fine-grained cell types in spatial transcriptomics. Nat Biotechnol. (2022) 40:661–71. doi: 10.1038/s41587-021-01139-4. PMID: 35027729

[20] Hanley CJ Waise S Ellis MJ Lopez MA Pun WY Taylor J . Single-cell analysis reveals prognostic fibroblast subpopulations linked to molecular and immunological subtypes of lung cancer. Nat Commun. (2023) 14:387. doi: 10.1038/s41467-023-35832-6. PMID: 36720863 PMC9889778

[21] Xu S Liu Y Ma H Fang S Wei S Li X . A novel signature integrated of immunoglobulin, glycosylation and anti-viral genes to predict prognosis for breast cancer. Front Genet. (2022) 13:834731. doi: 10.3389/fgene.2022.834731. PMID: 35432482 PMC9011196

[22] Forbord KM Lunde NN Bosnjak-Olsen T Johansen HT Solberg R Jafari A . Legumain is a paracrine regulator of osteoblast differentiation and mediates the inhibitory effect of TGF-β1 on osteoblast maturation. Front Endocrinol (Lausanne). (2024) 15:1445049. doi: 10.3389/fendo.2024.1445049. PMID: 39363898 PMC11446771

[23] Wang D Xiong M Chen C Du L Liu Z Shi Y . Legumain, an asparaginyl endopeptidase, mediates the effect of M2 macrophages on attenuating renal interstitial fibrosis in obstructive nephropathy. Kidney Int. (2018) 94:91–101. doi: 10.1016/j.kint.2017.12.025. PMID: 29656902

[24] Li H Chai X . PDPK1 governs macrophage M2 polarization via hypoxia-driven CD47/AKT-glycolytic Axis in endometriosis. Cell Signal. (2025) 134:111922. doi: 10.1016/j.cellsig.2025.111922. PMID: 40480430

[25] Li S Ding X Zhang H Ding Y Tan Q . IL-25 improves diabetic wound healing through stimulating M2 macrophage polarization and fibroblast activation. Int Immunopharmacol. (2022) 106:108605. doi: 10.1016/j.intimp.2022.108605. PMID: 35149293

[26] Cheng P Li S Chen H . Macrophages in lung injury, repair, and fibrosis. Cells. (2021) 10:436. doi: 10.3390/cells10020436. PMID: 33670759 PMC7923175

[27] Hou F Xiao K Tang L Xie L . Diversity of macrophages in lung homeostasis and diseases. Front Immunol. (2021) 12:753940. doi: 10.3389/fimmu.2021.753940. PMID: 34630433 PMC8500393

[28] Spatial transcriptomic characterization of pathologic niches in IPF. 10.1126/sciadv.adl5473 PMC11313858 39121212

[29] Qin X Lin X Liu L Li Y Li X Deng Z . Macrophage-derived exosomes mediate silica-induced pulmonary fibrosis by activating fibroblast in an endoplasmic reticulum stress-dependent manner. J Cell Mol Med. (2021) 25:4466–77. doi: 10.1111/jcmm.16524. PMID: 33834616 PMC8093963

[30] Liu F Yu F Lu YZ Cheng PP Liang LM Wang M . Crosstalk between pleural mesothelial cell and lung fibroblast contributes to pulmonary fibrosis. Biochim Biophys Acta Mol Cell Res. (2020) 1867:118806. doi: 10.1016/j.bbamcr.2020.118806. PMID: 32739525

[31] Shirahama-Noda K Yamamoto A Sugihara K Hashimoto N Asano M Nishimura M . Biosynthetic processing of cathepsins and lysosomal degradation are abolished in asparaginyl endopeptidase-deficient mice. J Biol Chem. (2003) 278:33194–9. doi: 10.1074/jbc.m302742200. PMID: 12775715

[32] Solberg R Lunde NN Forbord KM Okla M Kassem M Jafari A . The mammalian cysteine protease legumain in health and disease. Int J Mol Sci. (2022) 23:15983. doi: 10.3390/ijms232415983. PMID: 36555634 PMC9788469

[33] Jia D Chen S Bai P Luo C Liu J Sun A . Cardiac resident macrophage-derived legumain improves cardiac repair by promoting clearance and degradation of apoptotic cardiomyocytes after myocardial infarction. Circulation. (2022) 145:1542–56. doi: 10.1161/circulationaha.121.057549. PMID: 35430895

[34] Pei X Zhang SL Qiu BQ Zhang PF Liu TS Wang Y . Cancer cell secreted legumain promotes gastric cancer resistance to anti-PD-1 immunotherapy by enhancing macrophage M2 polarization. Pharm (Basel). (2024) 17:951. doi: 10.3390/ph17070951. PMID: 39065799 PMC11279811

[35] Pang L Guo S Khan F Dunterman M Ali H Liu Y . Hypoxia-driven protease legumain promotes immunosuppression in glioblastoma. Cell Rep Med. (2023) 4:101238. doi: 10.1016/j.xcrm.2023.101238. PMID: 37858339 PMC10694605

[36] Liu C Wang J Zheng Y Zhu Y Zhou Z Liu Z . Autocrine pro-legumain promotes breast cancer metastasis via binding to integrin αvβ3. Oncogene. (2022) 41:4091–103. doi: 10.1038/s41388-022-02409-4. PMID: 35854065

[37] Pan L Bai P Weng X Liu J Chen Y Chen S . Legumain is an endogenous modulator of integrin αvβ3 triggering vascular degeneration, dissection, and rupture. Circulation. (2022) 145:659–74. doi: 10.1161/circulationaha.121.056640. PMID: 35100526

[38] Shen L Kang L Wang D Xun J Chen C Du L . Legumain-deficient macrophages promote senescence of tumor cells by sustaining JAK1/STAT1 activation. Cancer Lett. (2020) 472:40–9. doi: 10.1016/j.canlet.2019.12.013. PMID: 31857155

[39] Arora S Dev K Agarwal B Das P Syed MA . Macrophages: Their role, activation and polarization in pulmonary diseases. Immunobiology. (2018) 223:383–96. doi: 10.1016/j.imbio.2017.11.001. PMID: 29146235 PMC7114886

[40] Zhang L Wang Y Wu G Xiong W Gu W Wang CY . Macrophages: friend or foe in idiopathic pulmonary fibrosis? Respir Res. (2018) 19:170. doi: 10.1186/s12931-018-0864-2. PMID: 30189872 PMC6127991

[41] Yang H Cheng H Dai R Shang L Zhang X Wen H . Macrophage polarization in tissue fibrosis. PeerJ. (2023) 11:e16092. doi: 10.7717/peerj.16092. PMID: 37849830 PMC10578305

[42] Yan L Wang J Cai X Liou YC Shen HM Hao J . Macrophage plasticity: signaling pathways, tissue repair, and regeneration. MedComm (2020). (2024) 5:e658. doi: 10.1002/mco2.658. PMID: 39092292 PMC11292402

[43] Rudnik M Hukara A Kocherova I Jordan S Schniering J Milleret V . Elevated fibronectin levels in profibrotic CD14(+) monocytes and CD14(+) macrophages in systemic sclerosis. Front Immunol. (2021) 12:642891. doi: 10.3389/fimmu.2021.642891. PMID: 34504485 PMC8421541

[44] Geervliet E Karkdijk E Bansal R . Inhibition of intrahepatic monocyte recruitment by Cenicriviroc and extracellular matrix degradation by MMP1 synergistically attenuate liver inflammation and fibrogenesis *in vivo*. Sci Rep. (2024) 14:16897. doi: 10.1038/s41598-024-67926-6. PMID: 39043893 PMC11266417

[45] Ratziu V Sanyal A Harrison SA Wong VW Francque S Goodman Z . Cenicriviroc treatment for adults with nonalcoholic steatohepatitis and fibrosis: final analysis of the phase 2b CENTAUR study. Hepatology. (2020) 72:892–905. doi: 10.1002/hep.31108. PMID: 31943293

[46] Francque SM Hodge A Boursier J Younes ZH Rodriguez-Araujo G Park GS . Phase 2, open-label, rollover study of cenicriviroc for liver fibrosis associated with metabolic dysfunction-associated steatohepatitis. Hepatol Commun. (2024) 8:e0335. doi: 10.1097/hc9.0000000000000335. PMID: 38285756 PMC10830067

[47] Landsman L Jung S . Lung macrophages serve as obligatory intermediate between blood monocytes and alveolar macrophages. J Immunol. (2007) 179:3488–94. doi: 10.4049/jimmunol.179.6.3488. PMID: 17785782

[48] Gu Y Lawrence T Mohamed R Liang Y Yahaya BH . The emerging roles of interstitial macrophages in pulmonary fibrosis: A perspective from scRNA-seq analyses. Front Immunol. (2022) 13:923235. doi: 10.3389/fimmu.2022.923235. PMID: 36211428 PMC9536737

[49] Aran D Looney AP Liu L Wu E Fong V Hsu A . Reference-based analysis of lung single-cell sequencing reveals a transitional profibrotic macrophage. Nat Immunol. (2019) 20:163–72. doi: 10.1038/s41590-018-0276-y. PMID: 30643263 PMC6340744

[50] Wendisch D Dietrich O Mari T von Stillfried S Ibarra IL Mittermaier M . SARS-CoV-2 infection triggers profibrotic macrophage responses and lung fibrosis. Cell. (2021) 184:6243–6261.e6227. doi: 10.1016/j.cell.2021.11.033. PMID: 34914922 PMC8626230

[51] Yu X Buttgereit A Lelios I Utz SG Cansever D Becher B . The cytokine TGF-β promotes the development and homeostasis of alveolar macrophages. Immunity. (2017) 47:903–912.e904. doi: 10.1016/j.immuni.2017.10.007. PMID: 29126797

