# Supplementary figures and images for: LGMN promotes crosstalk between macrophages and fibroblasts in pulmonary fibrosis: a potential therapeutic target
Source: Front Immunol. 2026 Apr 13;17:1789907. doi: 10.3389/fimmu.2026.1789907 (PMC13111070; doi:10.3389/fimmu.2026.1789907)

**Figure 8D**

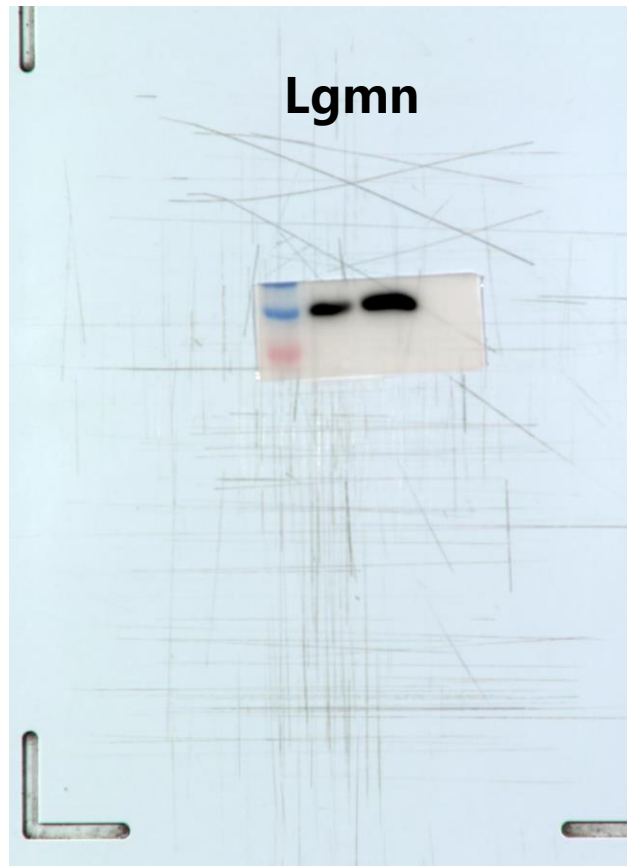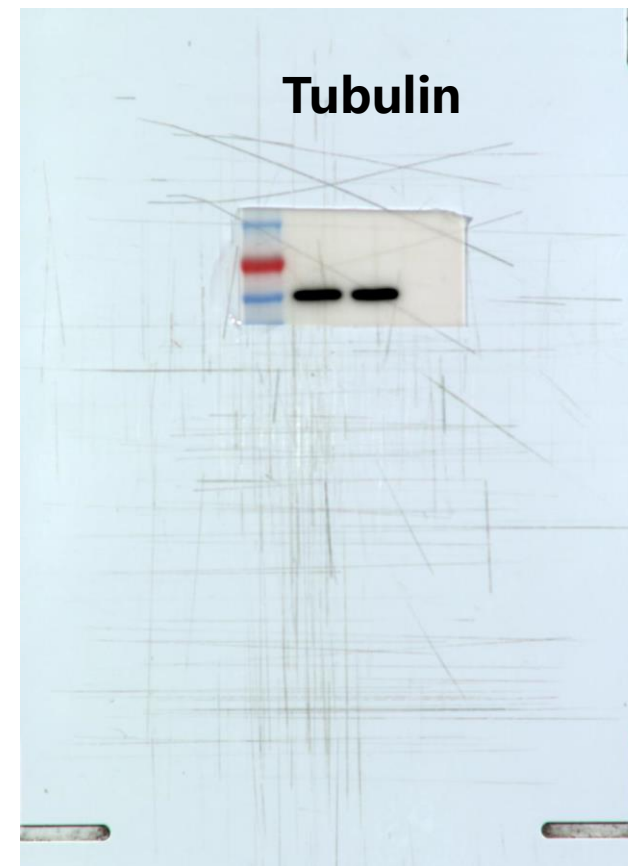

**Figure 8E**

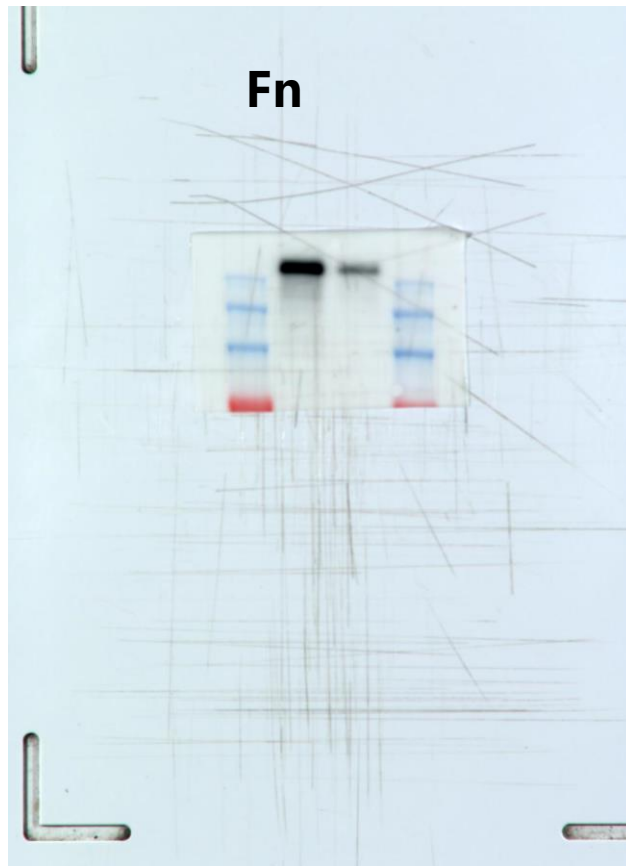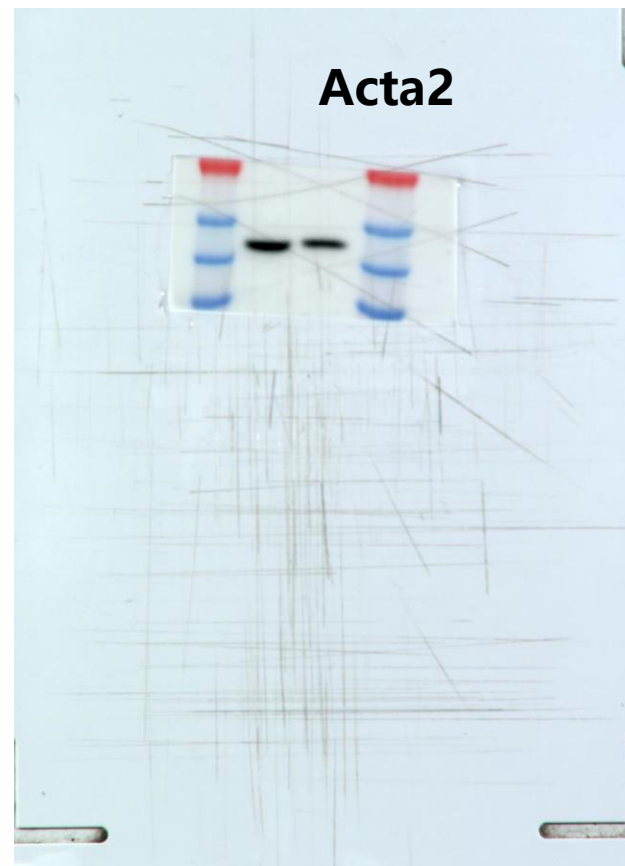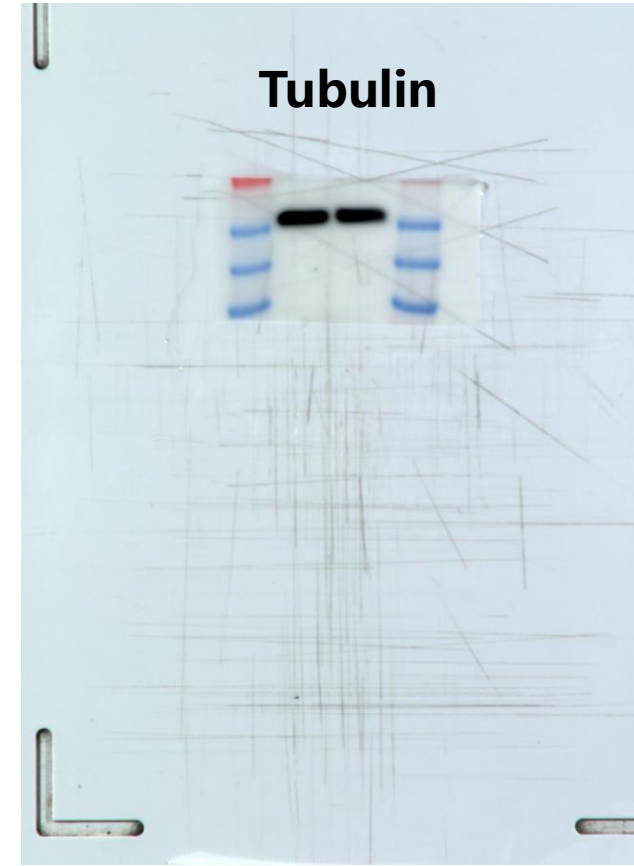

**Figure 8F**

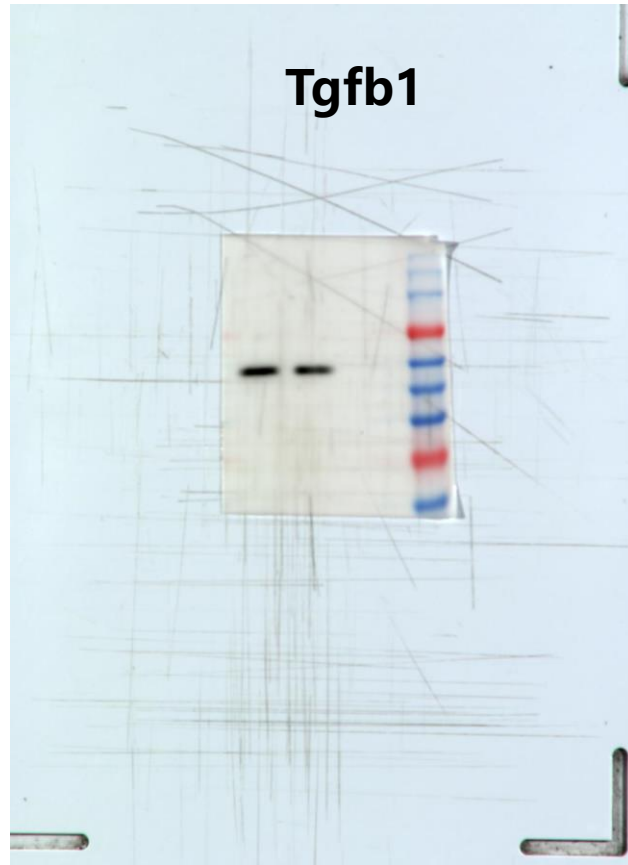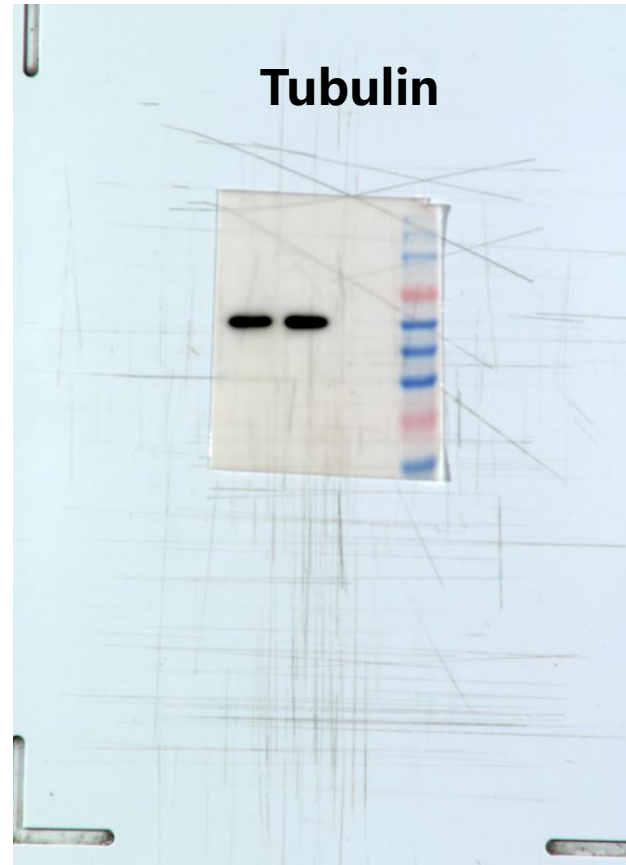

**Figure 8G**

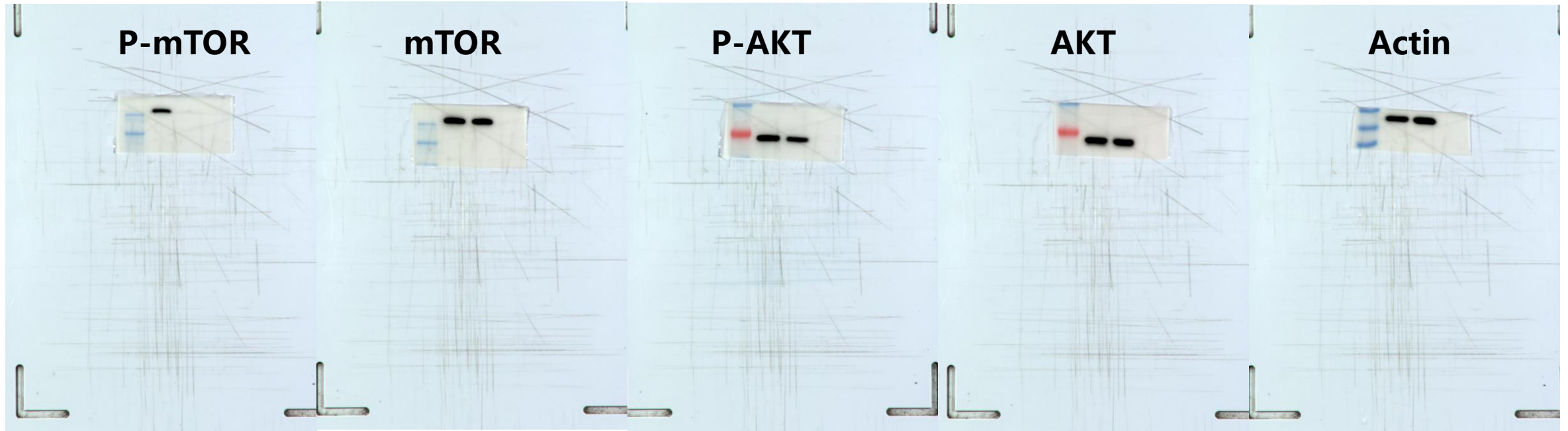

**Figure 9F**

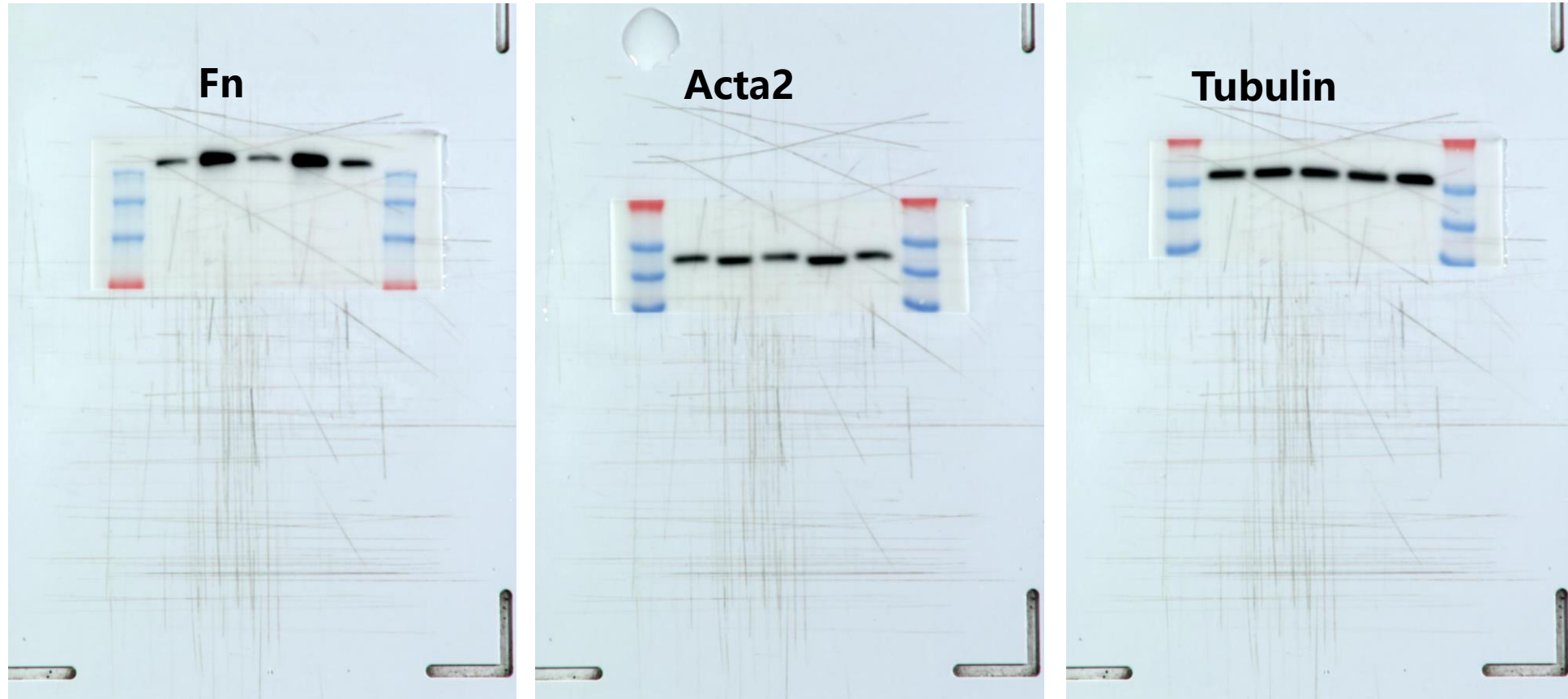

Supplement: Supplementary file 3 [file DataSheet3.pdf]

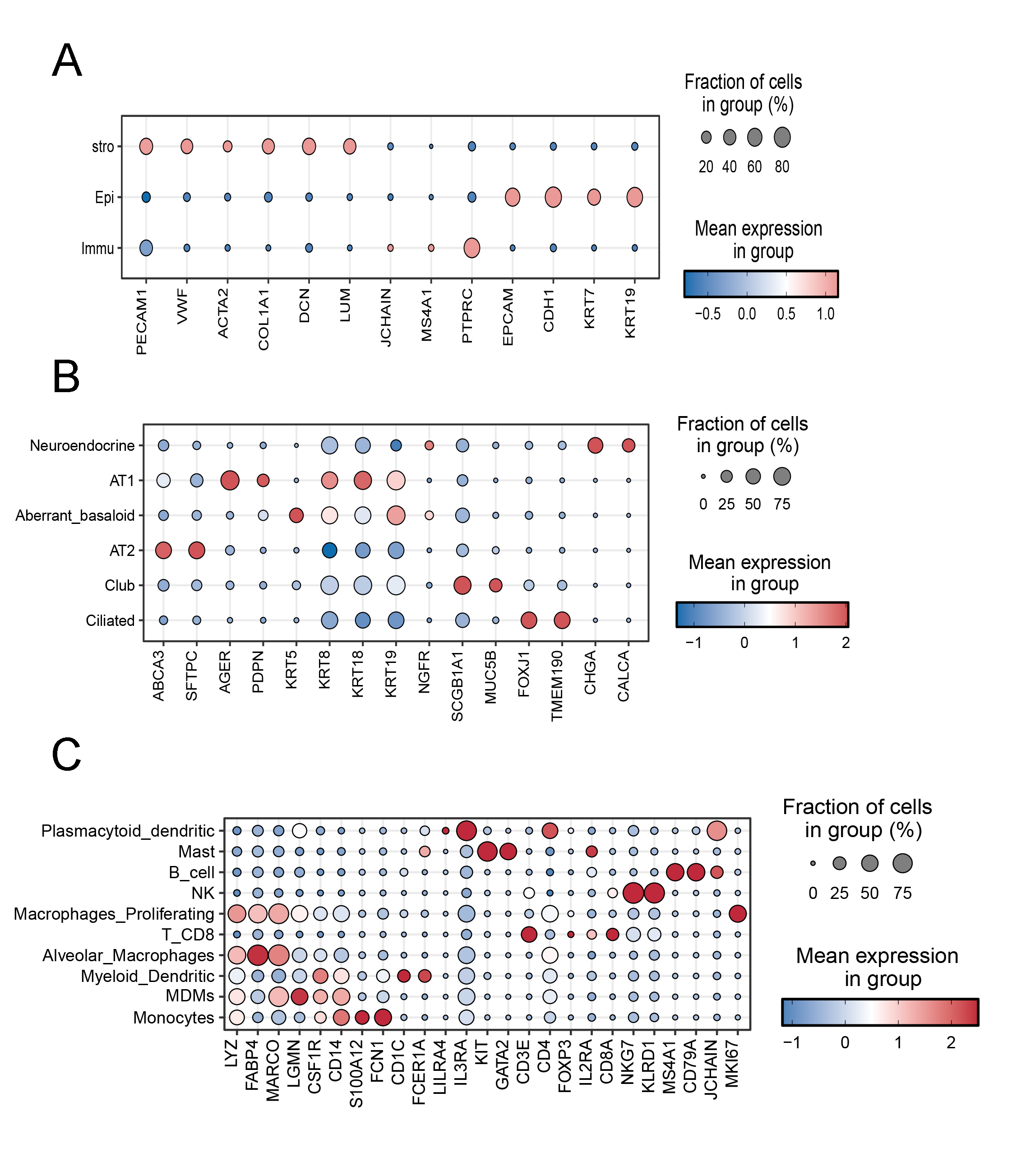

Supplement: Supplementary Figure 1 — (A) Average gene expression of selected marker genes for main cell clusters; (B) Average gene expression of selected marker genes for epithelial cell clusters; (C) Average gene expression of selected marker genes for immune cell clusters. [file Image1.tif]

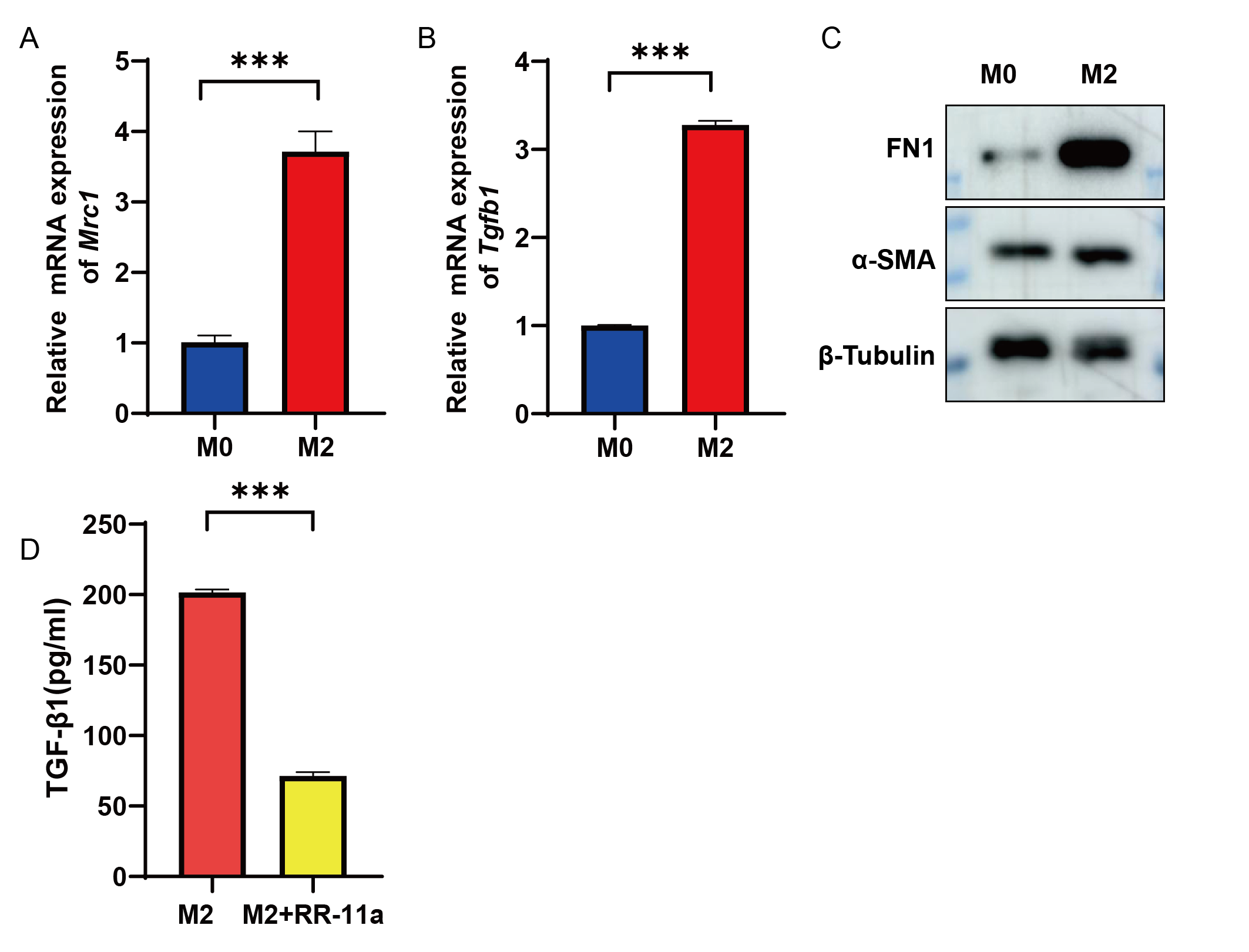

Supplement: Supplementary Figure 2 — (A) Relative mRNA expression of Mrc1 in Raw264.7 cells and M2 macrophages; (B) Relative mRNA expression of Tgfb1 in Raw264.7 cells and M2 macrophages; (C) supernatant from the M2 cultures induced the activation of NIH3T3 cells. (D) The supernatant of M2 cells was collected after treatment with RR-11a and ELISA performed for analysis of TGF-β1 secreted into the medium. [file Image2.tif]
