# Supplementary material for: LGMN promotes crosstalk between macrophages and fibroblasts in pulmonary fibrosis: a potential therapeutic target
Source: Front Immunol. 2026 Apr 13;17:1789907. doi: 10.3389/fimmu.2026.1789907 (PMC13111070; doi:10.3389/fimmu.2026.1789907)
Supplement: Supplementary file 4 [file Table1.docx]

Table S1: Primer sequences

| Cd206 R | ATGCCAAGTGGGAAAATCTG |  |  |
| --- | --- | --- | --- |
| Cd206 F | TGTAGCAGTGGCCTGCATAG |  |  |
| Tgfb1 F | GCAACAATTCCTGGCGTTA |  |  |
| Tgfb1 R | TTCCGTCTCCTTGGTTCAG |  |  |
| Actb F | GTGCTATGTTGCTCTAGACTTCG |  |  |
| Actb R | ATGCCACAGGATTCCATACC |  |  |
|  |  |  |  |
|  |  |  |  |
|  |  |  |  |
|  |  |  |  |
|  |  |  |  |
|  |  |  |  |
|  |  |  |  |
|  |  |  |  |
